# Reticular Chemistry: A Versatile Platform for Engineering Heterogenous Biocatalysts

**DOI:** 10.1002/advs.202519207

**Published:** 2025-12-12

**Authors:** Si Liu, Peiji Deng, Qianfan Chen, Qijun Sun, Yanhan Wang, Hongyu Shi, Kang Liang

**Affiliations:** ^1^ School of Chemical Engineering The University of New South Wales Sydney New South Wales 2052 Australia; ^2^ Australian Centre for NanoMedicine The University of New South Wales Sydney New South Wales 2052 Australia; ^3^ Graduate School of Biomedical Engineering The University of New South Wales Sydney New South Wales 2052 Australia

**Keywords:** biocatalysis, covalent organic frameworks, enzyme immobilization, hydrogen‐bonded organic frameworks, metal‐organic frameworks

## Abstract

Reticular materials, including metal‐organic frameworks (MOFs), covalent organic frameworks (COFs), and hydrogen‐bonded organic frameworks (HOFs), have emerged as a promising platform for enzyme immobilization due to their large surface area, tunable porosity, and diverse functional sites. However, the performance of enzymes encapsulated within these frameworks is frequently compromised, which primarily arises from spatial confinement, unfavorable interactions, and altered microenvironments that impair the native structure and dynamics of enzymes. A comprehensive understanding of the molecular events underlying enzyme encapsulation within frameworks is pivotal for the development of effective strategies to boost biocatalyst activity, thus unlocking its full potential in practical applications. Based on cutting‐edge examples, this review summarizes these approaches from the multiscale aspect, encompassing material tuning at the nano/macro level, interface design at the molecular interface level, and protein surface engineering at the molecular level. Meanwhile, the differences in improving the enzyme activity among MOFs‐, COFs‐, and HOFs‐based biocomposites are highlighted. Additionally, the regulations derived from the nano‐bio effect can achieve the nanobiohybrids with customized, non‐native biocatalytic functions, which are systematically discussed. Finally, the current challenges and opportunities in heterogeneous biocatalysts based on reticular chemistry are underscored, charting a path toward advanced designs and their translation into impactful real‐world applications.

## Graphical abstract

Rational design of reticular‐based heterogeneous biocatalysts enables significant enhancement in enzyme activity recovery and even achieves catalytic performance beyond native enzymes.

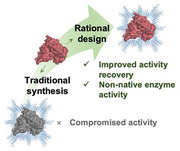

## 1 Introduction

Natural biological systems, as highly integrated and multifunctional networks, perform diverse functions across molecular, cellular, and organismal levels, including catalyzing biochemical reactions, transmitting signals, maintaining organization, adapting to environmental changes, and coordinating immune responses, functions essential for sustaining living organisms.^[^ ^1^, ^2^, ^3^, ^4^ ^]^ Among these processes, enzymes play a crucial role as highly specific functional biomacromolecules.^[^ ^5^, ^6^ ^]^ Moreover, enzyme‐catalyzed reactions can proceed under mild conditions and exhibit high catalytic efficiency unmatched by artificial catalysts.^[^ ^7^ ^]^ These merits make enzymes as powerful tools in practical applications, including industrial manufacturing, biosensor, environmental remediation, energy, and biomedical science.^[^ ^8^, ^9^, ^10^, ^11^, ^12^, ^13^, ^14^, ^15^ ^]^ Despite the various functional merits of enzymes, their practical application remains an on‐going challenge, primarily due to their intrinsic fragility under complex and harsh conditions, which limits their performance and aggravates the overall input cost.^[^ ^16^, ^17^ ^]^ Enzyme immobilization, meaning confining free enzymes within solid supports or defined microenvironments via physical or chemical strategies, represents one of the effective strategies to address the above issues.^[^ ^18^ ^]^ In nature, a similar strategy exists, where enzymes are housed within cellular architectures that offer protective microenvironments to safeguard the structural integrity and catalytic performance of enzymes under complex physiological conditions.^[^ ^19^, ^20^, ^21^, ^22^, ^23^, ^24^, ^25^, ^26^ ^]^ Inspired by this, encapsulating (referring to the physically confining or embedding processes) enzymes within a continuous matrix or porous host structure could efficiently improve the operational stability and sustainability of biocatalytic systems under diverse application conditions.^[^ ^27^, ^28^, ^29^, ^30^, ^31^ ^]^

Commonly used biocompatible supports for enzyme encapsulation include natural polymers,^[^ ^32^ ^]^ hydrogels,^[^ ^33^ ^]^ lipid‐based vesicles,^[^ ^34^ ^]^ and silica‐based porous materials.^[^ ^35^ ^]^ Despite enhanced biocatalysis durability using these materials, their practical applications remain constrained by limitations, such as low loading efficiency, inflexible pore architectures, poor mechanical stability, and mass‐transfer limitations.^[^ ^36^ ^]^ Hence, it is imperative to develop advanced immobilization platforms with well‐defined and tunable porosity, robust structural stability, and high enzyme accessibility. Metal‐organic frameworks (MOFs),^[^ ^29^, ^37^, ^38^, ^39^, ^40^, ^41^ ^]^ covalent organic frameworks (COFs),^[^ ^36^, ^42^, ^43^, ^44^ ^]^ and hydrogen‐bonded organic frameworks (HOFs),^[^ ^28^, ^45^, ^46^, ^47^, ^48^ ^]^ as emerging porous framework materials, have attracted increasing attention in enzyme immobilization. In contrast to traditional enzyme immobilization supports, MOFs, COFs, and HOFs exhibit precisely controlled pore structure, ultrahigh specific surface area, structural diversity, and facile functionalization, which is beneficial to facilitating efficient mass transfer, enhancing enzyme loading, and simplifying immobilization steps.^[^ ^36^, ^49^ ^]^ To date, extensive reviews have systematically summarized the enzyme immobilization methods employing these reticular frameworks, including surface bioconjugation (e.g., physical adsorption and covalent attachment), pore infiltration (e.g., diffusion into the pore channels of nanomaterials), and *in‐situ* encapsulation (e.g., biomimetic mineralization, co‐precipitation, and the latest solid‐state mechanochemical encapsulation).^[^ ^6^, ^28^, ^29^, ^31^, ^36^, ^44^, ^50^, ^51^, ^52^, ^53^, ^54^, ^55^, ^56^, ^57^ ^]^ Moreover, advanced characterization techniques for these nano‐biocomposites, such as solid‐state nuclear magnetic resonance, cryogenic electron microscopy, small‐angle X‐ray scattering, and spectroscopic analyses, have also been thoroughly reviewed in previous studies.^[^ ^44^, ^58^ ^]^ The constructed MOFs‐, COFs‐, and HOFs‐based immobilized enzymes have found broad applications in areas such as biocatalysis, biosensing, environmental remediation, drug delivery, and therapeutic systems.^[^ ^36^, ^44^, ^50^, ^53^, ^54^, ^55^, ^56^, ^57^, ^59^ ^]^

However, enzymes encapsulated within these reticular frameworks generally exhibit low catalytic activity recovery, greatly limiting their universal applicability across diverse applications.^[^ ^60^, ^61^, ^62^ ^]^ This limitation is primarily attributed to the impact of MOFs/COFs/HOFs on enzyme conformation and biocatalysis reaction dynamics.^[^ ^60^, ^61^, ^62^ ^]^ Specifically, spatial confinement, adverse interactions, or harsh synthesis conditions inherent to reticular chemistry could restrict structural flexibility and disrupt the native conformation of enzymes. Moreover, altered microenvironments, such as mass (e.g., substrates and cofactors) transfer limitations imposed by narrow pore channels, further hinder the efficient progression of enzymatic reactions within frameworks. Consequently, understanding the molecular events underlying enzyme encapsulation within reticular frameworks is crucial for advancing the methodology tuning nanobiocomposites with enhanced catalytic performance. Although Wang et al. first demonstrated in 2020 that engineering MOF‐biointerfaces could enhance enzyme activity,^[^ ^63^ ^]^ the past five years have witnessed remarkable advances in reticular chemistry‐based biocomposite systems, leading to diverse and more efficient strategies. Moreover, given the distinct assembly mechanisms and material properties of COFs and HOFs compared to MOFs, these regulatory principles are not directly transferable. Therefore, a comprehensive summary of recent developments is urgently needed to inform future design strategies.

**Scheme 1:**
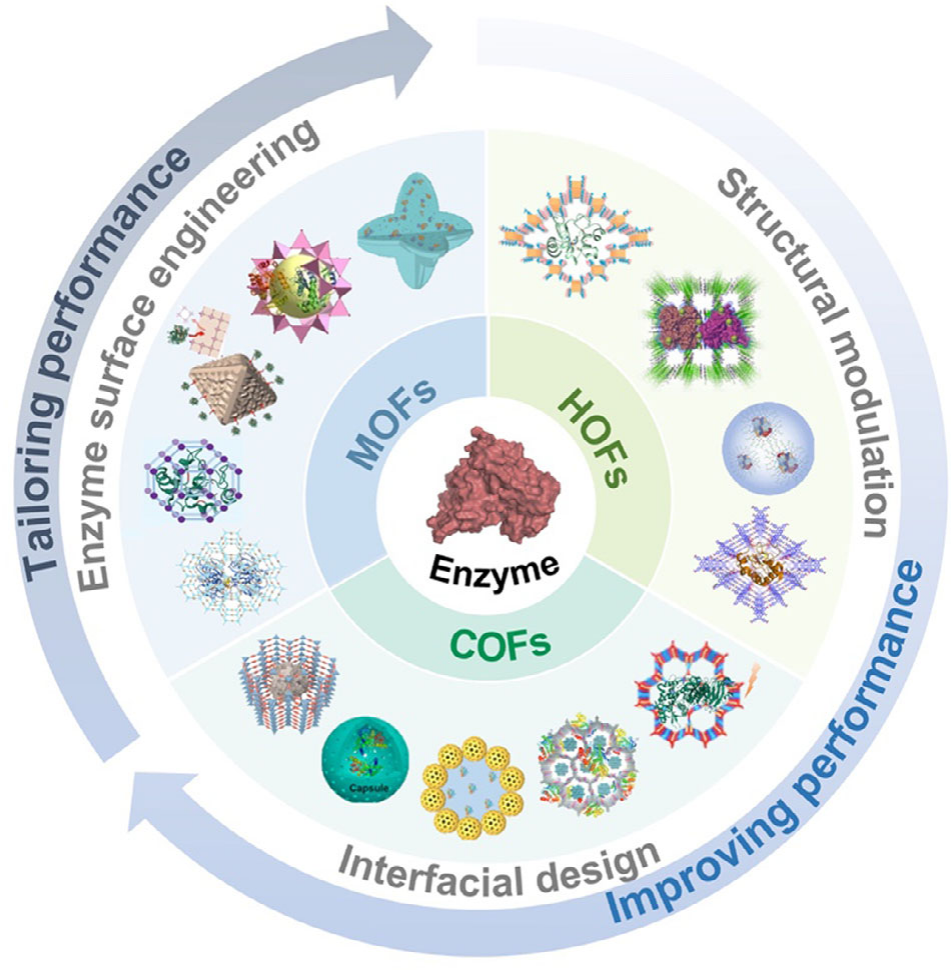
Schematic illustration of improving or tailoring the performance of enzyme‐MOF/COF/HOF composites.

**Scheme 2:**
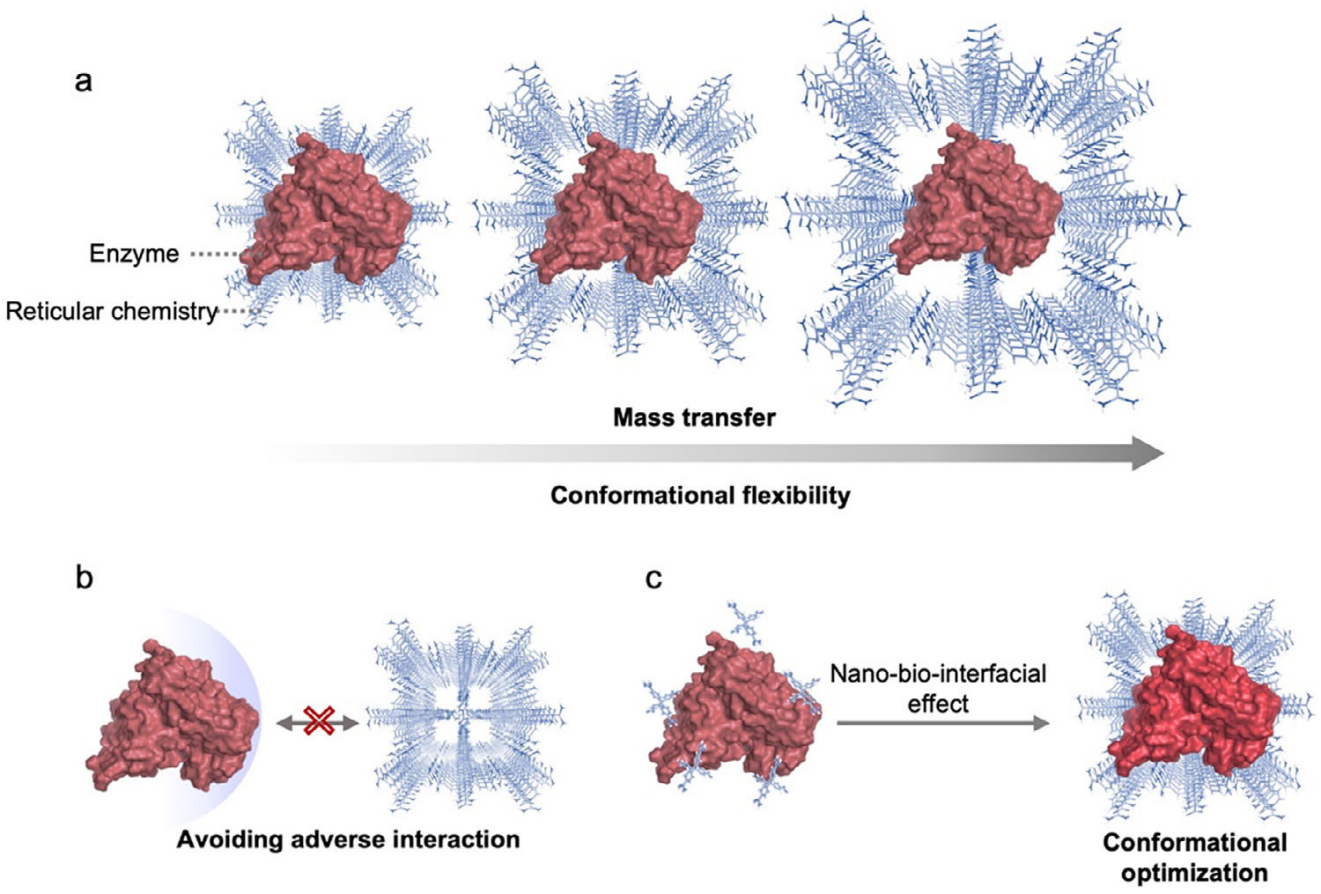
Schematic illustration of the performance‐tuning strategies for enzyme‐MOF/COF/HOF composites, including a) topology engineering of reticular chemistry, b) enzyme surface engineering, and nano‐bio‐interfacial interaction design.

Guided by molecular‐level insights, recent studies have sought to overcome the low activity recovery of nano–biohybrids via rational modulation of reticular framework topology, strategic nano‐bio interface design, and advanced enzyme surface engineering (**Scheme** **1**). This review provides a systematic summary of these approaches across nano‐ to molecular‐scale levels, drawing on state‐of‐the‐art research from the past five years (**Scheme** **2** and **Table** **1**). Given the distinct self‐assembly mechanisms of MOFs, COFs, and HOFs, the strategies are discussed separately to elucidate both shared principles and unique features. Furthermore, we highlight how these methods can endow biocomposites with functions beyond those of native enzymes (Scheme 1), underscoring the expanded role of reticular chemistry in nanobiocomposites beyond serving merely as carriers. Finally, the remaining challenges and future opportunities are discussed to provide insights into the intelligent construction of next‐generation frameworks‐based heterogeneous biocatalysts.

**Table 1:** Performance of reticular chemistry‐based biomcomposites tuned by different strategies.

Reticular framework		Biocatalysis system & Immobilization yield	Strategy	Catalytic performance	Stable/recyclable performance	Reference
**MOFs**	ZIF‐L	β‐Gal&Gox&HRP cascade (≈70%); NAD^+^ (72.6%)‐dependent GluDH (71.2%); NADH (83%)‐dependent FDH (76%)^a)^	Enlarging the pores via chemical etching	Up to a 16‐fold increase in enzymatic activity over the pristine biocatalytic ZIF‐L	Enhanced stability than the pristine biocatalytic ZIF‐L	[70]
	MIL‐88^b)^	Lipase (76.7 mg g^−1^)	Constructing mesoporous structures via a soft‐template method	Up to a 45‐fold increase in enzymatic activity over the pristine biocatalytic MIL‐88	Retaining 82.60% recovery after five consecutive batches	[71]
	ZIF‐8	Lipase (41.5% and 0.14 mg·mg^−1^)	Fabricating a hierarchically open‐capsule structure via a controlled self‐etching process	About three‐fold and 11.3‐fold increases in activity and catalytic efficiency over the unmodified ZIF‐8‐based biosystem and commercial Novozym 435, respectively	Retaining over 82% of initial catalytic activity after 5 cycles of reuse	[72]
	HKUST‐1; ZIF‐8; MIL‐88A	Lipase (121 mg g^−1^); GOx (81 mg g^−1^)	Constructing the single‐crystalline ordered macro‐microporous via etching polystyrene spheres	Up to a 45‐fold increase in enzymatic activity over the pristine biocatalytic MOFs	Almost unchanged enzymatic activity after 5 cycles of reuse	[73]
	ZIF‐8; ZIF‐67; HKUST‐1	Trypsin (325 µg mg^−1^)	Fabricating hierarchically porous MOFs by using polymer cubosomes as the sacrificial template	Up to 1.5‐fold and 2.8‐fold increases in BSA digestion efficiency over the corresponding free and ZIF‐8‐immobilized trypsin, respectively	Almost unchanged enzymatic activity after 5 cycles of reuse	[79]
	UiO‐66	Lipase (0.21 mg·g^−1^); CAT&GOx cascade system	Generating dynamic defects in MOFs by utilizing the dissociation equilibrium of MOFs mediated by enzymes	Excellent catalytic activity toward chiral resolution and hydrolysis reactions	Significantly enhanced stability; retaining over 95% of initial catalytic activity after 10 cycles of reuse	[81]
	PCN‐333	Cyt *c* (≈650 mg·g^−1^); HRP (≈730 mg·g^−1^); GOx (≈400 mg·g^−1^); PmHS2 (≈160 mg·g^−1^); Lipase (≈330 mg·g^−1^); glycoenzymes (containing 8 enzymes)	Engineering structure defects by using labile ligands	Up to a five‐fold increase in yield over the pristine PCN‐333‐based biosystem	Significantly enhanced stability under harsh conditions	[82]
	NU‐1000	Cyt *c* (6.4 wt %)	Nano‐bio‐interfacial effect	Up to a two‐fold increase in activity over free Cyt *c*	Thermal stability	[83]
	ZIF‐8	HRP (3.43 wt%)	Locking the active enzyme conformation by using MOF	Up to a 9.3‐fold increase in enzymatic activity over the conventionally prepared enzyme@ZIF‐8	Significantly enhanced stability; retaining over 77% of initial catalytic activity even after 5 cycles of reuse	[84]
	2D‐MOFs^c)^	Lipase (≈30–55 wt%)	Locking the active enzyme conformation by using MOF	Up to a 6.64‐fold increase in yield over native lipase	Retaining 85% of initial catalytic activity after 5 cycles of reuse	[85]
	NU‐1003	Lipase (12.51–17.45 wt%)	Tailoring the pore microenvironment to optimize enzyme conformation for maximal catalytic activity	About 1.57‐fold and 2.46‐fold higher activity than native lipase	Significantly enhanced stability; retaining more than 80% of initial catalytic activity after 4 cycles of reuse	[88]
	ZIF‐8	PEP^d)^ (‐)	Modulating the surface polarity of MOFs to stabilize the conformation of immobilized enzymes	Significant overpotential reduction of ≈81.6% and ≈86.2% compared to ZIF‐8@PEP and PEP, respectively	High operational stability	[86]
	MAF‐7; ZIF‐90	CAT (0.9–1.3 wt%)	Nano‐bio‐interfacial effect	Significantly increased catalytic activity over CAT@ZIF‐8	Enhanced stability; retaining more than 95% of initial catalytic activity after 10 cycles of reuse	[89]
	ZIFs	Lipase (0.17–0.36 wt%)	Nano‐bio‐interfacial effect	Up to a three‐fold increase in enzymatic activity over the free system	Enhanced stability	[90]
	MAF‐6^e)^	Esterase (3.1 wt%; 50.8%)	Activating the immobilized enzyme into its optimal conformation via the hydrophobic nature of MAF‐6	Up to a four‐fold increase in enzymatic activity over the free system	Enhanced stability; retaining more than 90% of initial catalytic activity after 5 cycles of reuse	[87]
	ZIF‐8	Cyt *c* (4.77 wt%); GOx (6.85 wt%); HRP (3.08 wt%)	Engineering enzyme surface residues to tune MOF nucleation and thus change the enzyme conformation	About a 19‐fold increase in activity over native Cyt *c*	Superior stability	[94]
**COFs**	TAPB‐DMTA‐COF^f)^	GOx (≈8.6 wt%); Cyt *c* (13.5 wt%); GOx (8.5 wt%)&HRP (4.8 wt%)cascade system	Preloading enzymes in low‐crystalline COF to pre‐protect enzyme activity	Up to a 4.7‐fold increase in activity over the free system; significantly enhanced enzyme activity over conventional encapsulation systems (negligible activity)	Retaining 80% of initial catalytic activity after 5 cycles of reuse	[98]
	PAH‐TAPB‐DMTA‐COF	NAD (25 µmol g^−1^)‐dependent ADH (150 mg g^−1^) &AmDH (10.7 mg g^−1^) cascade system; NADP (48.3%)‐dependent PAMO (78.1%)‐PTDH (86.5%)cascade system^g)^	Polyelectrolyte‐assisted pre‐protection	Achieving cofactor encapsulation in COFs; up to a two‐fold increase in activity over the biocatalytic TAPB‐DMTA COF	Enhanced stability; retaining over 70% of initial activity after 12 cycles of reuse	[99]
	TpDeth^h)^ COF	Lipase (11.5 wt %)	Biocompatible ionic liquid‐mediated COF growth for enzyme encapsulation	A 2.63‐fold increase in degradation rate over the poorly crystalline photo‐enzyme reactor	Excellent stability under harsh conditions	[100]
	COF‐LZU1; RT‐COF‐1; ACOF‐1^i)^	HRP (4.0–28.5 wt%); GOx (10.9 wt%); β‐Gal (10.1 wt%); Cyt *c* (15.7 wt%); Lipase (9.9 wt%)	In‐situ enzyme encapsulation in COFs under biofriendly conditions	Enhanced enzyme activity through weakening the enzyme‐COF interactions	Enhanced stability; retaining over 75% of initial activity after 12 cycles of reuse	[101]
	COF‐42; COF‐43	CAT (200 mg g^−1^); GOx; GOx&CAT cascade system; GOx&Hemin cascade system	Fabricating COF capsules via etching MOFs	Up to a 58% increase in activity over the pristine biocatalytic COFs	Almost unchanged enzymatic activity after 10 cycles of reuse	[102]
	TAPB‐DMTA‐COF	Lipase (≈102 mg g^−1^)	Fabricating hollow spherical COFs via an inside‐out Ostwald ripening process	About 1.6‐fold and 24.4‐fold increases in catalytic efficiency over lipase@COF (pristine) and free lipase, respectively	Enhanced stability; retaining over 82% of initial activity after 7 cycles of reuse	[103]
	TP‑TD^j)^‐COF	Cyt *c* (840 mg g^−1^)	Constructing hierarchically macro‐mesoporous COF via a sacrificial template method	About a 1.53‐fold increase in catalytic efficiency over the pristine biocatalytic COF	Enhanced stability; retaining over 80% of initial activity after 5 cycles of reuse	[104]
	TAPB‐BPTA^k)^‐COF	Lipase (28.4 wt%)	Fabricating COF‐based porous microcapsules via Pickering emulsion	Up to a 10.9‐fold increase in specific activity over the free system	Enhanced stability; retaining over 98% of initial activity after 6 cycles of reuse	[105]
	TAPB‐DMTA‐COF	Lipase (79.8 mg g^−1^)	Tailoring the pore microenvironment to optimize enzyme conformation	Up to a two‐fold increase in catalytic yield over the lipase in COF with other pore property	Retaining 68% of initial activity after 5 cycles of reuse	[107]
	NKCOF‐72; NKCOF‐73	Lipase (0.52 g g^−1^)	Tailoring COFs with photothermal conversion capability	About a 1.78‐fold increase in conversion over free lipase	Retaining over 80% of initial catalytic activity after 10 cycles of reuse	[108]
	TAPB‐DMTA‐COF	Lipase (16.4–29.1 wt%)	Encapsulating enzymes through a layer‐by‐layer assembly of COF nanosheets	About 1018‐fold and 254‐fold increases in turnover frequencies over free enzymes and COF powder biocomposites, respectively	Retaining over 90% of initial activity after an 80 h‐batched reaction	[109]
**HOFs**	HBF‐1; HBF‐2; HBF‐3	Cyt *c*; CAT; HRP; Myoglobin; Pepsin; GOx; Ovalbumin; Transferrin (24.3%–47.9%)	‐	Activity similar to or reduced compared to free enzymes	Enhanced stability; retaining over 90% of initial activity after 10 cycles of reuse	[61]
	SPF	α‐Amylase; Cellulase; Glucoamylase; Lipase; Proteinase K; DNA polymerase; CAT; GOx; Hemoglobin (over 40%)	‐	‐	Enhanced stability; retaining over 90% of initial activity after 10 cycles of reuse	[114]
	TNU‐14	α‐Amylase (17.9 wt%); CAT (17.5 wt%)	‐	‐	Enhanced stability; retaining over 69% of initial activity after 10 cycles of reuse	[115]
	TaTb nmHOF	LDH (≈98.0%); α‐Amylase; HRP	Designing nano‐sized mesoporous HOFs	Exhibiting comparable activity to the free enzyme	Enhanced stability; retaining over 90% of initial activity after 8 cycles of reuse	[117]
	PAH‐BioHOF‐1; PAH‐HOF‐101	NAD (38.6 mg g^−1^)‐dependent ADH (452.0 mg g^−1^) &AmDH (20.0 mg g^−1^) cascade system; NADP (20 mg g^−1^)‐dependent PAMO (78 mg g^−1^) &PTDH (93 mg g^−1^) cascade system	Polyelectrolyte‐assisted co‐encapsulation	First achieving the co‐encapsulation of enzymes and a cofactor in HOFs; up to 60‐fold and 1.2‐fold increases in cascade activity over corresponding free and BioHOF‐1 systems, respectively	Enhanced stability; retaining over 89% of initial cascade activity after 12 cycles of reuse	[116]
	HOF‐PTBA	Cyt *c* (40.8 wt%)	Increasing pore size	Enhanced protein loading	Enhanced stability; retaining 71% of initial cascade activity after 10 cycles of reuse	[120]
	BioHOF‐1	DAAO (500 mg g^−1^)	Protein surface engineering	About a 6.5‐fold increase in specific activity compared to the pristine DAAO in BioHOF‐1	Enhanced stability; retaining over 80% of initial cascade activity after 10 cycles of reuse	[121]
	BioHOF‐1	CAT (58 wt%)	Vapor‐assisted mechanochemical synthesis	Up to a ≈3‐fold increase in catalytic yield over the encapsulated CAT prepared in solution	Retaining over 75% of initial cascade activity after 8 cycles of reuse	[124]
	HOF‐101	Cyt *c* (40.85 wt%)	Tailoring the pore microenvironment to optimize enzyme conformation	About a 4.9‐fold increase in conversion compared to the native Cyt *c*	Enhanced stability; retaining over 80% of initial cascade activity after 5 cycles of reuse	[125]
	HOF‐101	Cyt *c* (39 wt%)	Using the nano‐biointerface interaction between HOF and enzyme	Achieving non‐native CAT‐like activity	Enhanced stability; retaining about 70% CAT‐like activity after 7 cycles of reuse	[126]

^a)^ β‐Gal, β‐galactosidase; GluDH, glutamate dehydrogenase; FDH, formate dehydrogenase;

^b)^ MIL‐88, Materials of Institut Lavoisier‐88;

^c)^ 2D‐MOFs were synthesized by the coordination interaction between Zn^2+^/Cu^2+^ and 2,3,6,7,10,11‐triphenylenehexol;

^d)^ PEP, Pepsin;

^e)^ MAF‐6, Metal–Azolate Framework‐6;

^f)^ TAPB, 1,3,5‐tris(4‐aminophenyl)benzene; DMTA, 2,5‐dimethoxyterephthalaldehyde;

^g)^ PAMO, phenylacetone monooxygenase; PTDH, phosphite dehydrogenase;

^h)^ Tp, 1,3,5‐triformylphloroglucinol; Deth, 2,5‐diethoxyterephthalohydrazide;

^i)^ RT, room temperature; ACOF‐1, azine‐linked COF‐1;

^j)^ TD, 4,4′,4″‐ (1,3,5‐triazine‐2,4,6‐triyl)trianiline;

^k)^ BPTA, 2,5‐bis(4‐aminophenyl)terephthalamide.

## 2 MOFs‐Based Biocomposites

### 2.1 Insight into MOFs‐Based Biocomposites

MOFs are assembled from organic linkers and metal‐containing clusters or ions.^[^ ^64^, ^65^, ^66^ ^]^ Compared with conventional nano‐ and mesoporous materials, MOFs offer several remarkable advantages, including ultrahigh porosity, tunable pore sizes, customizable composition and architecture, high guest‐loading capacity, and a large internal surface area.^[^ ^64^, ^65^, ^66^ ^]^ These properties render MOFs an attractive platform for enzyme immobilization. Enzymes can be confined within MOFs via *in‐situ* encapsulation during framework formation (e.g., biomimietic mineralization), or through post‐synthetic approaches (e.g., pore infiltration).^[^ ^6^ ^]^ However, the predominantly microporous nature of conventional MOFs (typically <2 nm, e.g., zeolitic imidazolate framework‐8 (ZIF‐8),^[^ ^67^ ^]^ University of Oslo‐66 (UiO‐66),^[^ ^68^ ^]^ and Hong Kong University of Science and Technology‐1 (HKUST‐1))^[^ ^69^ ^]^ presents challenges for enzyme immobilization via post‐infiltration and hinders efficient mass transfer of biocatalysis, thus compromising the catalytic performance of enzymes within MOFs. Moreover, these MOFs with microporous structures confine enzymes in a compact state, reducing their conformational flexibility and consequently diminishing their catalytic activity. The interfacial interactions between MOFs and enzymes can also affect the enzyme conformation organization.

### 2.2 Topology Tuning of MOFs

**Figure 1:**
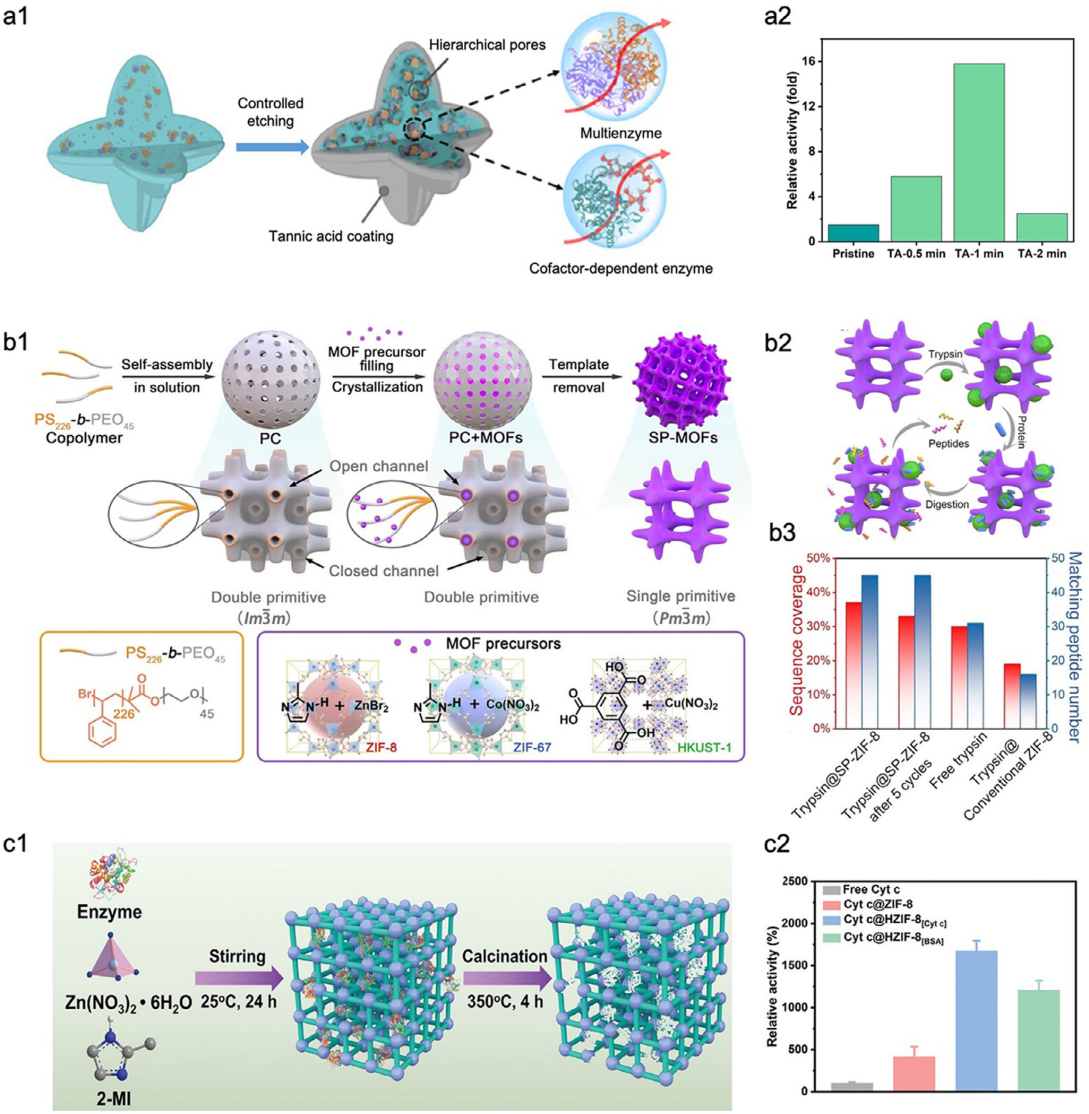
Representative examples of enlarging the pores of MOFs‐based biocomposites at the macroscopic level. a1) Schematic of the construction of a hierarchically porous biocatalytic MOF microreactor via a TA etching strategy. a2) Relative activity of three‐enzyme microreactors synthesized after TA etching at different times. Reproduced with permission.^[^ ^70^ ^]^ Copyright 2023, Wiley‐VCH. b1) Schematic illustration of structural defect formation in MOF via a sacrificial templating strategy, in which the template is based on a cubosome‐forming lyotropic liquid crystal phase. b2) Schematic illustration of trypsin immobilization and BSA digestion in the 3D continuous channels of SP‐ZIF‐8. b3) Digestion of BSA by trypsin@SP‐ZIF‐8, free trypsin, and trypsin@ZIF‐8. Reproduced with permission.^[^ ^79^ ^]^ Copyright 2023, Wiley‐VCH. c1) Schematic of depicting the process for hierarchically porous ZIF‐8 through a “physical imprinting” strategy. c2) The relative activity of cytochrome *c* (Cyt *c*) at different forms. Reproduced with permission.^[^ ^80^ ^]^ Copyright 2024, Wiley‐VCH.

Design of mesoporous or hierarchically porous MOFs is an effective strategy for improving enzyme immobilization and facilitating mass transfer. In parallel, such porous architectures can improve the conformational flexibility and active‐site accessibility of encapsulated enzymes by alleviating interfacial constraints at the MOF‐enzyme interface. All these advantages collectively contribute to the enhanced catalytic activity of the encapsulated enzymes. A straightforward approach to enlarging the pores of MOFs involves chemical etching^[^ ^70^, ^71^, ^72^ ^]^ and the introduction of sacrificial templates,^[^ ^73^, ^74^, ^75^ ^]^ both at the macroscopic level. Our group previously developed a versatile strategy for constructing hierarchically porous ZIF‐L via tannic acid (TA)‐mediated etching (**Figure** **1**a1).^[^ ^70^ ^]^ Owing to its weak acidity, TA would release protons in a controlled manner, leading to the formation of mesopores within the interior of ZIF‐L. Meanwhile, TA could adsorb onto the external surface of ZIF‐L to form a stable layer, which is beneficial to preserve the structural integrity and robustness of ZIF‐L. The co‐encapsulated multienzyme system in ZIF‐L displayed significantly enhanced cascade activity after TA etching, with up to a 16‐fold increase in catalytic performance (Figure 1a2). This result demonstrates that engineering enlarged pores in MOFs through defect formation can greatly improve the catalytic efficiency of immobilized enzymes. Moreover, the constructed ZIF‐L‐TA could co‐encapsulate different nicotinamide adenine dinucleotide (NAD^+^)‐dependent biocatalytic systems, beneficial to enhancing the accessibility of enzymes to cofactors, thus improving the whole catalytic efficiency of biosystems. It should be emphasized that precise control over the etching time is essential since excessive etching may cause substantial framework collapse and serious cofactor leakage. Li et al. developed hierarchically mesoporous zirconium (Zr)‐based frameworks by precisely tuning the torsional angles of organic linkers to achieve the co‐encapsulation of multienzymes and cofactors.^[^ ^76^ ^]^ The hierarchical pore system allowed enzymes to reside in large cavities, while smaller pores and inter‐channel voids accommodated NAD(P) and substrates. Such an architecture endowed biomolecules with enhanced conformational flexibility, albeit accompanied by partial NAD(P) loss. Alternatively, the cofactors can be immobilized onto polymer backbones via physical adsorption^[^ ^77^ ^]^ or covalent linkage^[^ ^78^ ^]^ before co‐precipitation, allowing their simultaneous encapsulation with multiple enzymes within MOFs.

In addition to the *in‐situ* etching strategy,^[^ ^70^, ^71^, ^72^ ^]^ sacrificial templating (i.e., polystyrene microspheres,^[^ ^73^, ^74^ ^]^ emulsions,^[^ ^75^ ^]^ and surfactants^[^ ^75^ ^]^) has emerged as another widely used and versatile approach for constructing meso‐ and macro‐porous MOFs. Recently, Li et al. presented an innovative approach to fabricating hierarchically porous MOFs by utilizing polymer cubosomes (PCs) as the sacrificial template (Figure 1b).^[^ ^79^ ^]^ Specifically, a double primitive (DP)‐structured PC was first constructed through the solution self‐assembly of a simple linear polystyrene‐*block*‐poly(ethylene oxide) (PS‐*b*‐PEO) amphiphilic block copolymer. Due to the existence of a three‐dimensional (3D) continuous open channel in PC, MOF precursors easily diffused into PC and replicated the single network of DP structure through the crystallization, thus forming the PC+MOFs composite structure. The single‐network MOFs (SP‐MOFs) cubosomes were ultimately obtained by removing the PC template via solvent extraction under mild conditions (Figure 1b1). This strategy has demonstrated the applicational feasibility in different MOFs, including ZIF‐8, ZIF‐67, and HKUST‐1, which opens a new avenue for controlling the overall morphology and hierarchically porous structure of MOFs. SP‐ZIF‐8 was employed to encapsulate trypsin to evaluate the advantage of its hierarchically porous structure for the biocatalytic reaction (Figure 1b2). The results represented that the proteolysis efficiency of trypsin@SP‐ZIF‐8 was indeed higher than that of trypsin@ZIF‐8 and even exceeded that of free trypsin (Figure 1b3). This excellent performance was attributed to two key structural features: 1) the 3D continuous mesochannels, which shortened diffusion distances and enhanced molecular mobility, thus promoting enzyme‐substrate contact; 2) the large mesopores, which can accommodate the bulky substrate molecule (bovine serum albumin, BSA), enabling efficient catalysis within the porous matrix. To better integrate enzymes with MOFs, Liu et al. proposed a “physical imprinting” approach in which the target enzyme served as a sacrificial template to fabricate size‐ and shape‐specific cavities within the MOF framework for precise enzyme encapsulation (Figure 1c1).^[^ ^80^ ^]^ This structural matching strategy not only ensured a tight spatial fit between the enzyme and the MOF matrix but also minimized conformational distortion upon immobilization. As a result, the confined enzymes within these MOFs exhibited up to 1670% higher relative activity compared to the corresponding free enzymes (Figure 1c2). Notably, the substantial performance enhancement also arises from the nanoconfinement‐induced structural reconfiguration of enzyme molecules, underscoring the importance of appropriately regulating the conformation of confined enzymes for optimal functionality.

**Figure 2:**
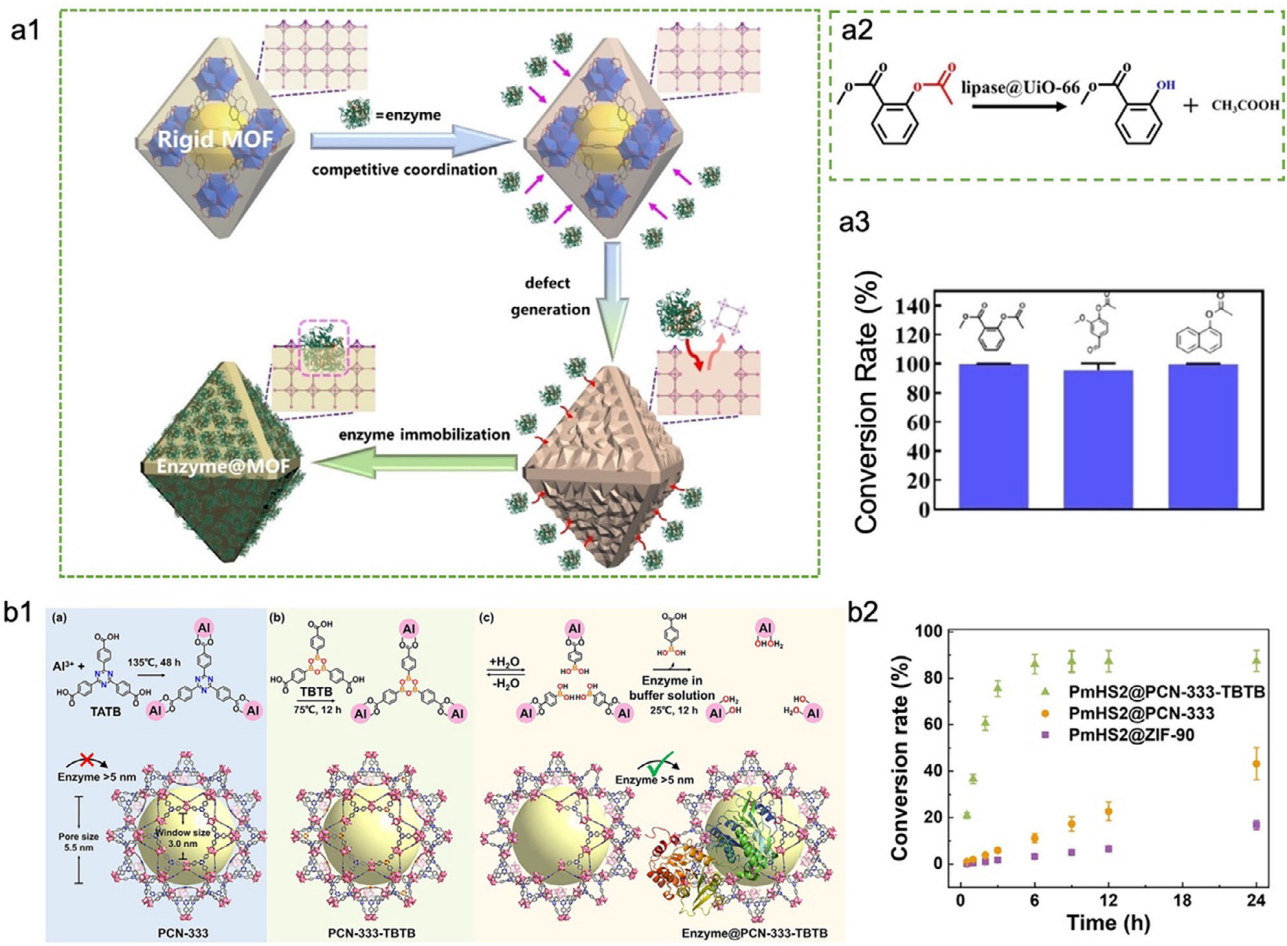
Representative examples of enlarging the pores of MOFs‐based biocomposites at the microscopic level. a1) Schematic illustration of a dynamic defect generation strategy for enzyme immobilization based on the dissociation equilibrium of MOFs. a2) Reaction equation of the hydrolysis of AME. a3) Conversion of lipase@UiO‐66 catalyzing different substrates. Reproduced with permission.^[^ ^81^ ^]^ Copyright 2023, Wiley‐VCH. b1) Schematic illustration of a dynamic defect‐generation strategy for enzyme immobilization via the incorporation of labile boroxine linkers into the mesoporous PCN‐333. b2) Catalytic performance of PmHS2‐encapsulated PCN‐333‐TBTB, PCN‐333, and ZIF‐90. Reproduced with permission.^[^ ^82^ ^]^ Copyright 2024, Wiley‐VCH.

Compared to macroscopic regulation, constructing structural defects through tuning the crystallization assembly process of nano‐bio hybrids offers a more precise, controllable strategy for synthesizing hierarchically porous MOFs. Chen and co‐workers reported a dynamic defect‐generation strategy to enhance the structural flexibility of encapsulated enzyme and facilitate mass transfer in MOFs (**Figure** **2**a1).^[^ ^81^ ^]^ In this approach, the enzyme itself served as a macromolecular ligand to modulate the coordination environment of MOF. Specifically, the nitrogen‐rich surface of the enzyme competed with organic linkers for coordination to metal nodes, thus inducing partial dissociation of the crystalline structure of MOF and generating structural defects at the enzyme‐MOF interface. This self‐regulated process enabled the gradual internalization of the enzyme into the MOF matrix, resulting in efficient immobilization. Using encapsulated lipase (lipase@UiO‐66) as an example, this dynamic defect‐engineering strategy could achieve not only high immobilization efficiency but also a hydrolysis yield exceeding 99% for aspirin methyl ester (AME) (Figure 2a2,a3). In addition to AME, lipase@UiO‐66 also demonstrated excellent hydrolytic activity toward two other substrates with a bigger molecule size (Figure 2a3). These results highlight the effectiveness of enzyme‐induced defect formation for constructing functional biocatalytic MOFs with enhanced activity, improved substrate accessibility, and excellent structural adaptability. Dynamic linker dissociation represents another effective strategy for creating structural defects in MOFs at the microscopic level. Recently, Wang and co‐workers designed a mesoporous MOF by incorporating a labile tritopic linker containing boroxine moieties (1,3,5‐tris(4‐boronic acid phenyl)benzene, TBTB), as one of the organic building block (Figure 2b1).^[^ ^82^ ^]^ TBTB undergoes hydrolysis under aqueous conditions (Figure 2b1, centre), and the extent of its dissociation can be precisely tuned by adjusting the water concentration in its surrounding environment. In contrast to TBTB, the co‐linker (4,4′,4′’‐s‐triazine‐2,4,6‐triyl‐tribenzoate, TATB) exhibits extremely stable under the same conditions. Therefore, the resulting MOF (porous coordination network‐333 (PCN‐333)‐TBTB), assembled from both TBTB and TATB organic linkers, developed localized structural defects while retaining long‐range crystallinity and structural robustness. Moreover, the controlled linker dissociation effectively unlocked previously inaccessible pore space within PCN‐333, enabling the successful encapsulation of *Pasteurella multocida* heparosan synthase 2 (PmHS2), a large enzyme with dimensions of 9.8 × 6.5 × 4.7 nm (Figure 2b1, right). Notably, the catalytic performance of PmHS2@PCN‐333‐TBTB far exceeded that of PmHS2@PCN‐333 and PmHS2@ZIF‐90 (Figure 2b2), which is due to the enhanced conformational flexibility of the enzyme and improved mass transfer conferred by the internal structural defects in PCN‐333‐TBTB. These findings collectively indicate that designing hierarchically porous MOFs is conductive to enhancing the mass transport and conformational flexibility of enzymes, thus recapitulating the characteristics of native biocatalytic systems and improving the enzyme activity recovery.

### 2.3 Enzyme‐MOFs Interface Design

**Figure 3:**
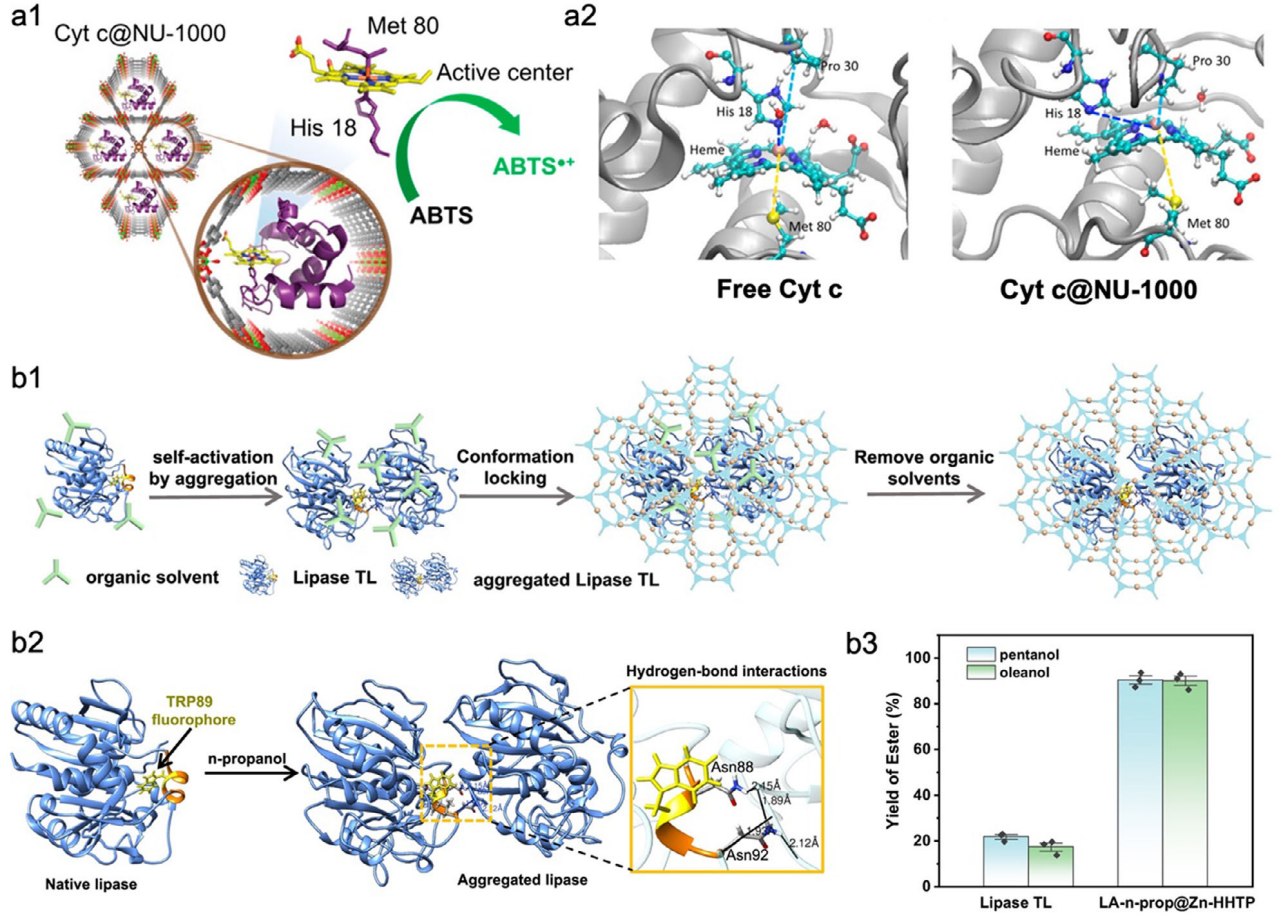
Representative examples of enhancing the performance of MOFs‐based biocomposites through the MOFs‐enzyme interface. a1) Schematic illustration of Cyt *c* encapsulated within NU‐1000 and its oxidation of ABTS. a2) The configurations around heme of Cyt *c* in water (left) and inside MOF NU‐1000 (right). Reproduced with permission.^[^ ^83^ ^]^ Copyright 2020, American Chemical Society. b1) Schematic illustration of the conformation locking of self‐activated lipase TL aggregates by encapsulating them within MOF. b2) Structural opening of the active‐pocket lid in lipase TL induced by *n*‐propanol treatment. b3) Comparison of product yields catalyzed by free lipase TL and LA‐n‐prop@Zn‐HHTP across different substrates. Reproduced with permission.^[^ ^85^ ^]^ Copyright 2025, Springer nature.

Despite these benefits of the topology modulation strategy, the improvement in enzyme activity generally remains confined to levels comparable to those of native enzymes. Chen et al. found that cytochrome *c* (Cyt *c*) exhibited enhanced catalytic activity upon encapsulation in Northwestern University‐1000 (NU‐1000) compared with free Cyt *c* (**Figure** **3**a1).^[^ ^83^ ^]^ Further analysis revealed that this improvement resulted from the opening of the enzyme's active center (Figure 3a2), induced by hydrophilic/hydrophobic interactions between the organic linker and the enzyme. This finding highlights that the interplay between MOFs and enzymes can induce favorable enzyme conformations, thus facilitating the bioactivity. However, such activity enhancement is enzyme‐dependent due to variations in protein conformation and interfacial interactions. Therefore, for the majority of enzymes, rational design of MOF–enzyme interfacial interactions guided by the enzyme's structure–function relationship is essential, which has the potential to reshape enzyme conformational landscapes, thus empowering immobilized enzymes with catalytic performance that exceeds their native counterparts. Liang et al. employed the ultrasound (US) pretreatment to tune the conformation of horseradish peroxidase (HRP) into a catalytically optimal state, maximizing its catalytic activity.^[^ ^84^ ^]^ The US‐activated HRP was subsequently encapsulated in situ within ZIF‐8. Due to the interfacial interactions between US‐HRP and ZIF‐8, the catalytically optimal conformation of HRP was effectively locked. Accordingly, the resulting US‐HRP@ZIF‐8 exhibited a 5.3‐fold enhancement in activity compared to free HRP without US treatment. This significant improvement underscores the effectiveness of leveraging nano‐bio interface effects to lock enzymes in catalytically favourable states, which provides a versatile strategy for enhancing the performance of enzyme‐MOF biocomposites. Recently, Huang et al.^[^ ^85^ ^]^ reported a similar approach in which lipase from *Thermomyces lanuginosus* (TL) was pretreated with polar organic solvents (i.e., *n*‐propanol and isopropanol) to induce enzyme aggregation and open the active‐site lid (Figure 3b1,b2). The activated enzyme aggregates were then encapsulated within a 2D MOF (Zn‐2,3,6,7,10,11‐hexahydroxytriphenylene framework, Zn‐HHTP), effectively “locking” the optimized conformation of lipase TL (Figure 3b1, centre). Notably, once encapsulated, the favourable enzyme conformation was stably retained even after the removal of the organic solvent activators (Figure 3b1, right). As a result, the resulting biocatalyst (LA‐n‐prop@Zn‐HHTP) achieved over 80% yield in catalyzing reactions with pentanol or oleanol, significantly outperforming the 28% yield obtained with the native lipase TL (Figure 3b3). These studies collectively emphasize the importance of enzyme pre‐activation in attaining conformationally optimized states prior to encapsulation and subsequently locking these states within the framework. Nevertheless, this locking method primarily relies on pre‐activation rather than the nano‐bio interfacial interactions to tune enzyme activity.

**Figure 4:**
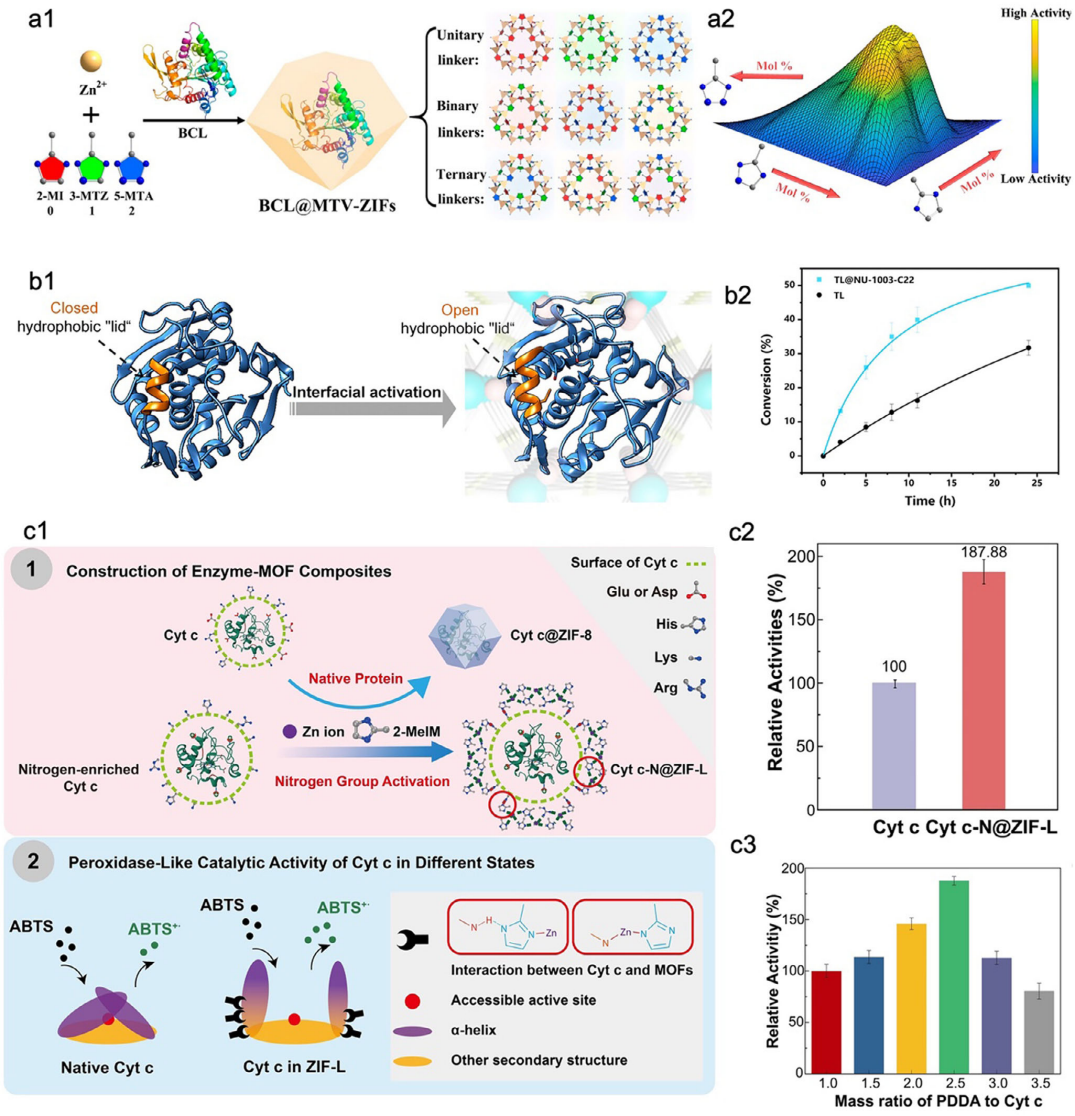
Representative examples of enhancing the performance of MOFs‐based biocomposites through regulating the MOFs‐enzyme interface. a1) Schematic illustration of encapsulating BCL in multivariate ZIFs. a2) Activity change of BCL with altering the ligand of ZIF‐8. Reproduced with permission.^[^ ^90^ ^]^ Copyright 2021, American Chemical Society. b1) Schematic illustration of the conformational change in the active site of lipase TL induced by the interfacial activation on fatty‐acid‐modified NU‐1003. b2) Time‐dependent conversion rates of free and immobilized lipase TL. Reproduced with permission.^[^ ^88^ ^]^ Copyright 2024, Springer nature. c1) Schematic illustration of the MOF structure transformation induced by the encapsulation of nitrogen‐enriched Cyt *c* (top) and the catalytic site exposure of encapsulated Cyt *c* due to its interface interaction with MOF (bottom). c2) Relative activity of Cyt *c* and Cyt *c*‐N@ZIF‐L. c3) Effect of the mass ratio of PDDA and Cyt *c* on the catalytic activity of Cyt *c*‐N@ZIF‐L. Reproduced with permission.^[^ ^94^ ^]^ Copyright 2025, Wiley‐VCH.

Insights into the enzyme conformation‐function relationship enable the rational design of MOF pore properties to fine‐tune enzyme‐MOF interfacial interactions, ultimately boosting enzymatic performance.^[^ ^86^, ^87^, ^88^ ^]^ Most enzymes maintain their native conformation in aqueous environments; however, exposure to hydrophobic surroundings often perturbs their tertiary structure, resulting in loss of catalytic activity. Given this, Liang et al. utilized hydrophilic metal–azolate framework‐7 (MAF‐7) to encapsulate catalase, which retained a significant degree of enzymatic activity.^[^ ^89^ ^]^ In contrast, the hydrophobic ZIF‐8 perturbed the native enzyme conformation, thus resulting in a complete loss of catalytic activity for the encapsulated catalase. This pioneering study demonstrated that the key role of the pore microenvironment in MOFs in regulating enzyme conformation, thus greatly advancing the future development of activity‐beneficial reticular frameworks. Li et al. reported multivariate MOFs by changing the functionality and ratio of linkers to tune and optimize the interior environment of MOFs, ultimately enhancing the bioactivity of encapsulated *Burkholderia cepacia* lipase (BCL) (**Figure** **4**a1).^[^ ^90^ ^]^ In BCL, the helical lid exhibits a hydrophilic exterior and a hydrophobic interior. The addition of nonpolar solvents to the aqueous solution of free BCL induces lid opening at the interface, increasing the exposure of hydrophobic regions and the active site to substrates, which in turn enhances the lipase activity (Figure 4a2). Therefore, enhancing the hydrophilicity within the ZIF‐8 microenvironment could open the catalytic pocket of BCL through hydrogen (H)‐bonding interactions between its lid and the noncoordinated nitrogen (N) atoms of ZIF, thus improving the bioactivity. However, further increasing hydrophilicity and excessive H‐bonds would disrupt the optimal conformation of BCL, ultimately resulting in decreased enzymatic activity. Moreover, the spatial configuration of N atoms in ZIFs cooperated with H‐bonding interactions to modulate enzyme activity, highlighting a distinctive advantage of reticular chemistry over traditional methods for activating the catalytic pocket of lipases. Benefiting from the simplicity of multivariate MOFs synthesis (notably multi‐ligand MOFs), this concept has been extensively applied to modulate enzymatic activity and further integrated with pore‐engineering strategies to maximize the catalytic efficiency of enzymes confined within MOFs.^[^ ^91^, ^92^ ^]^ Furthermore, multivariate MOFs with multiple metal centers are favorable for enhancing the activity of metalloenzymes by facilitating their substrate affinity.^[^ ^93^ ^]^

Regulating the pore channel environment of MOFs through ligand engineering is another effective strategy to tailor enzyme activity according to the nano‐bio‐interface effect. For example, Guo et al. utilized fatty acids with varying alkyl chain lengths to precisely modify the mesoporous channels of NU‐1003, thus creating a hydrophobic environment within the framework pores.^[^ ^88^ ^]^ This modification could open the active‐site lid of lipase TL through interfacial activation induced by the hydrophobic pore microenvironment of NU‐1003 (Figure 4b1). Notably, lipase TL was selectively encapsulated within the mesopores of NU‐1003, while its micropores remained accessible to substrates. This strategic combination of surface hydrophobization and compartmentalization not only stabilized the catalytically active conformation of lipase TL but also facilitated mass transfer efficiency. Accordingly, the immobilized lipase TL exhibited a 1.57‐fold increase in ester hydrolysis activity compared with the free enzyme (Figure 4b2).

Moving beyond the design of MOF pore architectures, enzyme engineering via precise tailoring of surface residues emerges as another powerful strategy to tailor MOF‐enzyme interfacial interactions. Wu et al. recently designed a favorable interaction between Cyt *c* and MOF through the targeted modification of enzyme molecule (Figure 4c).^[^ ^94^ ^]^ Specifically, Cyt *c* was first modified with a cationic polymer, poly(diallyldimethylammonium chloride (PDDA), to neutralize surface carboxylate groups and increase the exposure of amine functionalities. The nitrogen‐rich Cyt *c* was then introduced into the ZIF‐8 growth solution. During MOF crystallization, the nitrogen‐rich patches on Cyt *c* competed with 2‐methylimidazolate ligands for coordination to Zn(II), steering the reticular framework assembly away from the dense ZIF‐8 topology toward the more open, leaf‐like ZIF‐L architecture (Figure 4c1, top). This interfacial modulation not only enlarged the accessible pore space but also induced rearrangement of the secondary structure of Cyt *c*, leading to greater exposure of the heme active site (Figure 4c1, bottom). As a result, PDDA‐modified Cyt *c* encapsulated in ZIF‐L (denoted Cyt *c*‐N@ZIF‐L) displayed a 1.9‐fold increase in specific activity compared to native Cyt *c* (Figure 4c2). Noted that the catalytic activity of Cyt *c*‐N@ZIF‐L increased progressively with higher PDDA concentrations (PDDA:Cyt *c* = 1.0–2.5) (Figure 4c3), suggesting that the elevated nitrogen levels could promote stronger interfacial interactions. This result further confirms that intelligent regulation of the nano‐bio‐interface effect could confer enzymes with superior conformational states, thus achieving higher activity over native biocatalytic systems. Nevertheless, this rational design remains challenging for most biocatalytic systems. To address this, Wang et al. reported a pre‐protection method in which a histidine‐based shell was created around the enzymes before MOF encapsulation, thereby preventing adverse interactions with MOF building units.^[^ ^95^ ^]^ Compared with conventionally encapsulated enzymes, those immobilized using this pre‐protection approach exhibited significantly enhanced catalytic efficiency. Moreover, the versatility of this strategy across different MOFs suggests that enzyme engineering‐induced pre‐protection may offer broader applicability than precise interface design.

## 3 COFs‐Based Biocomposites

### 3.1 Insights into the COFs‐Based Biocomposites

**Figure 5:**
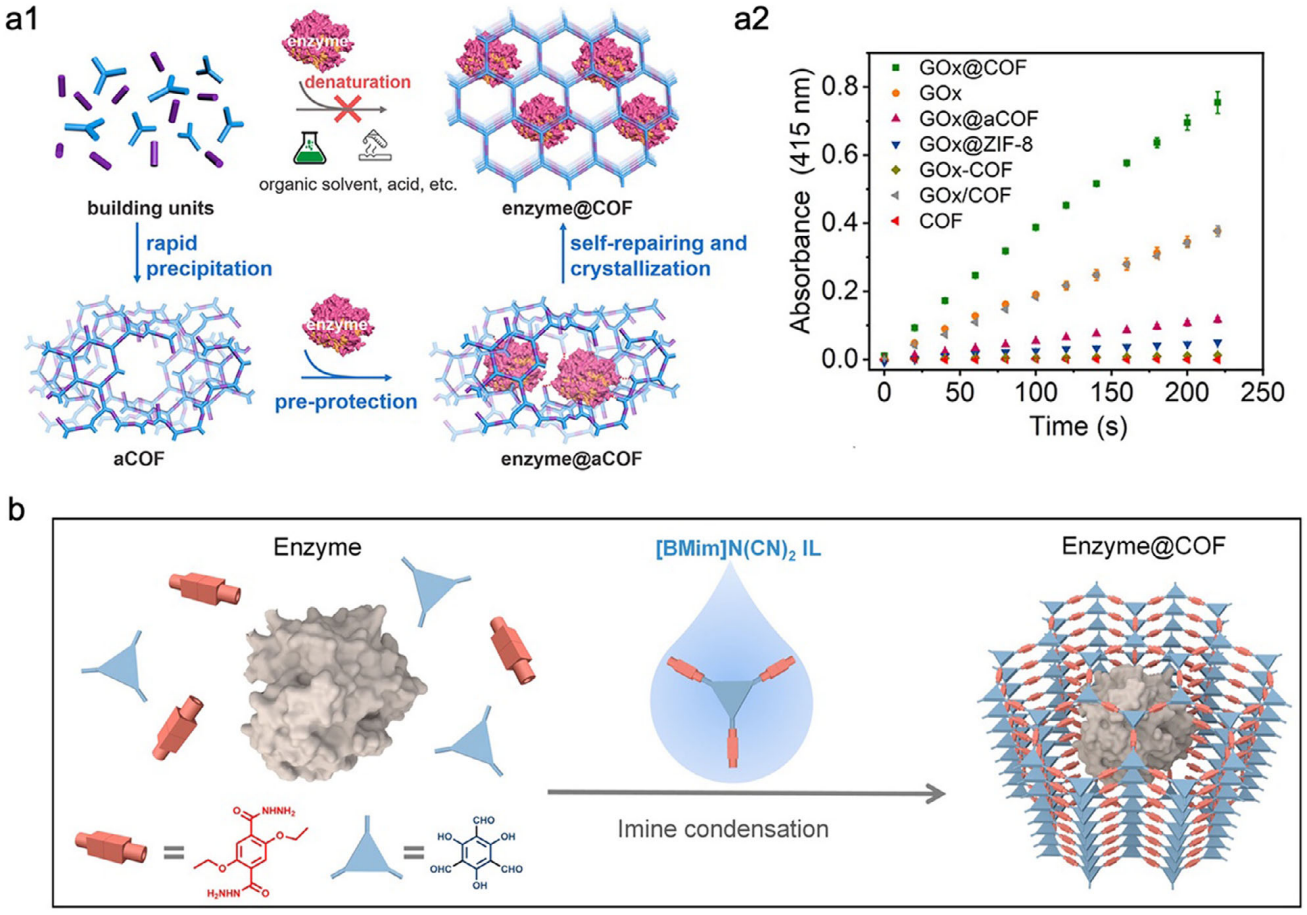
Representative examples of enhancing the performance of COFs‐based biocomposites through mitigating the detrimental effects of COFs synthesis conditions. a1) Schematic illustration of a pre‐protection strategy for enzyme encapsulation in COF via a self‐repairing and crystallization process. a2) Time‐dependent catalytic curves of free GOx, pristine COF, and GOx immobilized at different forms. Reproduced with permission.^[^ ^98^ ^]^ Copyright 2023, American Chemical Society. b1) Schematic representation of the ionic liquid‐assisted synthesis of crystalline enzyme@COF biocatalysts via dynamic polymerization. Reproduced with permission.^[^ ^100^ ^]^ Copyright 2024, Wiley‐VCH.

COFs are a class of porous materials constructed via covalent bonds, characterized by high crystallinity, uniform pore size distribution, and excellent thermal stability, making them ideal candidates for use as capsules in enzyme encapsulation.^[^ ^44^, ^96^ ^]^ However, the synthesis of COFs typically requires harsh conditions, such as the use of organic solvents, acids, bases, or high temperatures, which may lead to enzyme inactivation during the encapsulation process.^[^ ^97^ ^]^ In recent years, researchers have made preliminary explorations into bio‐friendly strategies for the in‐situ encapsulation of enzymes in COFs. For instance, Zhang et al. reported an innovative in situ encapsulation strategy for enzyme–COF biocomposites by harnessing the self‐repairing and crystallization properties of dynamic covalent polymerization (**Figure** **5**a1).^[^ ^98^ ^]^ In this approach, glucose oxidase (GOx) was initially embedded within a low‐crystallinity COF (aCOF) under mild conditions. Subsequently, the intermediate network underwent a self‐repairing process, gradually transforming into a highly crystalline COF structure. Due to the protective effect of aCOF on GOx, the resulting GOx@COF displayed significantly higher catalytic activity than the co‐precipitated counterpart (GOx‐COF), as shown in Figure 5a2. Furthermore, GOx@COF also exhibited superior performance compared to free Gox (Figure 5a2), which may be due to favorable confinement effects, enhanced mass transfer, and stabilization of the enzyme microenvironment afforded by the dynamic polymerization and crystallization processes. While this approach facilitates the retention of enzyme activity, it still faces limitations in its applicability to other enzymes. This is likely due to the fact that the second crystallization step still relies on the use of high concentrations of acetic acid to induce COF formation. To address this constraint, Li et al. developed an improved strategy.^[^ ^99^ ^]^ Specifically, a protective shell was constructed around the enzyme via electrostatic interactions between the enzyme surface and a polyelectrolyte. This polyelectrolyte layer not only effectively protected the enzyme from harsh synthetic conditions but also helped preserve its native conformation. Moreover, the NAD(P)H cofactors could be electrostatically adsorbed onto the polyelectrolyte, enabling their co‐encapsulation with multienzymes within the COF matrix. This strategy demonstrated broad versatility across two distinct NAD(P)H‐dependent multienzyme cascade systems, exhibiting excellent stability and recyclability. Nonetheless, the overall preparation process remains tedious and time‐consuming.

Gao et al. introduced a one‐pot, aqueous‐phase, ionic liquid–mediated dynamic polymerization method for fabricating highly crystalline enzyme–COF biocatalysts under mild and biocompatible conditions (Figure 5b).^[^ ^100^ ^]^ By adding a trace amount of an imidazolium‐based ionic liquid, the COF network underwent reversible bond formation, enabling the in situ self‐assembly of a crystalline COF “exoskeleton” that directly encapsulated the enzyme, without the need for harsh solvents or extreme pH conditions. Similarly, Liang et al. developed a one‐pot synthetic strategy for constructing enzyme–COF biocomposites in aqueous media, completing the entire encapsulation process within 10–20 min.^[^ ^101^ ^]^ By comparing three different COFs systems, they found that HRP@COF‐LZU1 (COF‐Lanzhou University 1) exhibited the highest catalytic activity, which was attributed to the weakest interaction between COF‐LZU1 and host enzyme. This relatively weak interaction was beneficial to preserving the native conformation of the enzyme, thus enhancing the activity retention. These findings offer valuable insights into how interfacial interactions affect the bioactivity of in situ–encapsulated enzyme–COF biocomposites.

### 3.2 Topology Tuning of COFs

**Figure 6:**
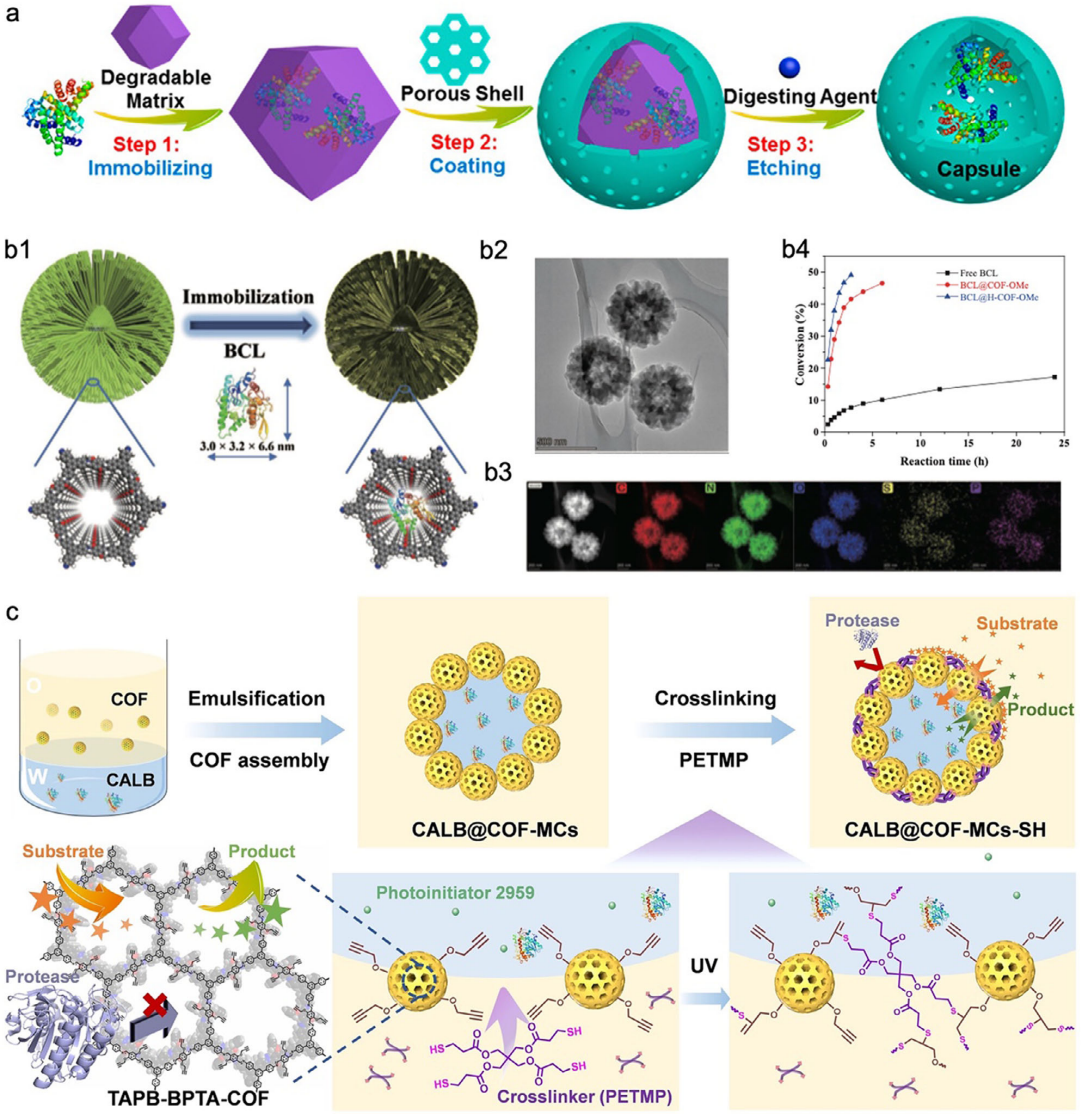
Representative examples of enhancing the performance of COFs‐based biocomposites through mitigating the COFs‐enzymes interface interactions. a) Schematic illustration of the fabrication of enzyme@COF capsules by etching the MOF core. Reproduced with permission.^[^ ^102^ ^]^ Copyright 2020, Wiley‐VCH. b1) Schematic representation of BCL immobilization on the hollow spherical H‐COF‐OMe. b2) Transmission electron microscopy and b3) elemental mapping of BCL@H‐COF‐OMe. b4) Conversion of 1‐phenyl ethanol with free BCL, BCL@COF‐OMe, and BCL@H‐COF‐OMe. Reproduced with permission.^[^ ^103^ ^]^ Copyright 2023, Springer nature. c) Schematic illustration of the construction of CALB@COF‐MCs‐SH porous microreactors with crosslinked COF shells based on Pickering emulsion. Reproduced with permission.^[^ ^105^ ^]^ Copyright 2023, Wiley‐VCH.

Given the influence of the nano–bio interface on the activity of encapsulated enzymes, weakening the interaction between COFs and enzymes is also crucial for recovering the conformational flexibility of enzymes and achieving high activity recovery. Owing to the inherently rigid and robust nature of COF frameworks, the design of hollow COF structures has emerged as an effective strategy for accommodating enzymes. In recent years, a variety of hollow COF architectures have been developed for enzyme encapsulation. For example, Li et al. reported a versatile sacrificial templating approach employing MOFs as removable templates to create hollow COF capsules specifically tailored for enzyme immobilization (**Figure** **6**a).^[^ ^102^ ^]^ In this strategy, enzymes were first encapsulated within ZIF‐90, forming enzyme@ZIF‐90 composites that served both as protective reservoirs and sacrificial templates. Subsequently, a COF‐42‐B shell was grown around the ZIF‐90 core via heterogeneous nucleation and localized dynamic covalent polymerization, yielding enzyme@ZIF‐90@COF‐42‐B core–shell structures. The MOF core was then selectively removed under mild acidic conditions, leaving behind hollow COF‐42‐B capsules that provide a spacious internal environment for the encapsulated enzymes. This strategy proved effective across various biocatalytic systems, where enzymes immobilized within COF‐42‐B capsules exhibited significantly enhanced catalytic performance compared to those directly embedded in bulk COF‐42‐B. These results further highlight the importance of maintaining conformational flexibility when constructing enzyme‐reticular framework biocomposites. Zhao et al. developed a distinct method for synthesizing hollow spherical COFs (H‑COF‑OMe) via an inside‐out Ostwald ripening mechanism under mild, ambient conditions, aimed at immobilizing BCL (Figure 6b1).^[^ ^103^ ^]^ Initially, uniform solid COF spheres were formed, followed by dissolution of the internal crystallites and their outward redeposition, ultimately generating hollow structures with crystalline shells that retained high surface area, crystallinity, and structural integrity (Figure 6b2,b3). The resulting biocatalyst, BCL@H‑COF‑OMe, exhibited superior performance in the kinetic resolution of secondary alcohols compared to both non‐hollow COF supports (BCL@COF‑OMe) and free BCL (Figure 6b4). This enhanced activity was primarily attributed to the spacious and hydrophobic microenvironment inside the hollow COF cavity, which conferred high conformational flexibility and a favorable catalytic microenvironment for the encapsulated enzyme. Compared with the template‐mediated strategies for constructing COF capsules,^[^ ^102^, ^104^ ^]^ this template‐free approach based on Ostwald ripening offers several advantages, including simplified synthesis, elimination of sacrificial core materials, and formation of well‐defined hollow structures under mild conditions. However, the lack of precise spatial control over enzyme positioning and potential variability in cavity size may limit its general applicability, especially for multi‐enzyme systems or size‐sensitive biocatalysis.

To overcome these challenges, COFs have also been employed as building blocks for constructing microcapsules. Feng et al. designed a robust COF‐based microcapsule (COF‐MCs‐SH) via Pickering emulsion templating and thiol‐ene crosslinking, which enabled the encapsulation of *Candida antarctica* lipase B (CALB) within an internal aqueous microenvironment (Figure 6c).^[^ ^105^ ^]^ Compared to single‐shell hollow COFs, the COF‐MCs‐SH structure offered a more spacious and substrate‐accessible interior, thereby more effectively facilitating biocatalytic reactions. Moreover, the unique architecture and physicochemical properties of COF‐MCs‐SH endowed the system with substrate selectivity: the microporous COF shell excluded non‐target substrates via size exclusion, while enriching target molecules through hydrophobic interactions. These synergistic features collectively contributed to the outstanding esterification performance of CALB@COF‐MCs‐SH, which even surpassed that of the free enzyme.

### 3.3 Enzyme‐COFs Interface Design

**Figure 7:**
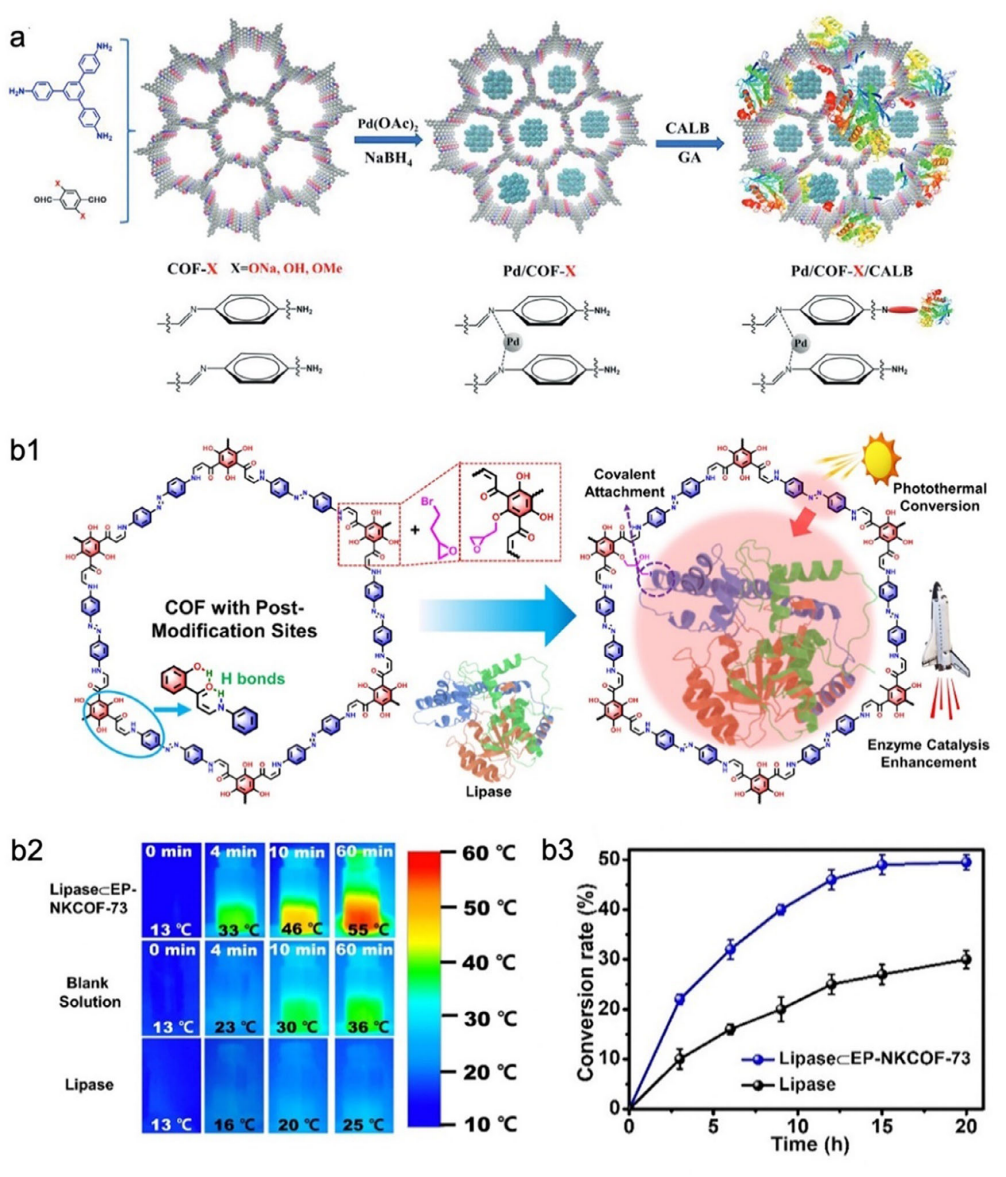
Representative examples of regulating the performance of COFs‐based biocomposites. a1) Schematic illustration of molecular engineering strategies for tuning COF structures to optimize metal–enzyme cascade catalysis. a2) Cascade yield of co‐encapsulated CALB and Pt in COFs with different tectons. Reproduced with permission.^[^ ^107^ ^]^ Copyright 2024, Wiley‐VCH. b1) Schematic illustration of the photothermal‐promoted strategy for enhancing the activity of lipase within COF. b2) Thermal images of different systems. b3) The conversion rate curves of free lipase and lipase@EP‐NKCOF‐73 under light. NK, Nankai University. Reproduced with permission.^[^ ^108^ ^]^ Copyright 2024, Wiley‐VCH.

As previously discussed, increasing the conformational flexibility of enzyme molecules is beneficial for improving the activity recovery of enzymes encapsulated within COFs. However, it is worth noting that reducing enzyme–framework interactions does not necessarily correlate positively with enhanced enzymatic activity.^[^ ^106^ ^]^ In contrast, the rational regulation of nano–bio interfacial interactions can, in some cases, yield catalytic activities that are difficult to achieve with free enzyme systems, which is like MOFs‐based biocomposites. Zhao et al. employed the molecular engineering strategy to tailor the functional groups of COF, specifically sodium oxide form (─ONa), hydroxyl group (─OH), and methoxy group (─OMe), resulting in structural microenvironments with highly polar (hydrophilic), moderately polar, and weakly polar (hydrophobic) characteristics, respectively (**Figure** **7**a1).^[^ ^107^ ^]^ These functionalized COFs were utilized to co‐immobilize palladium nanoparticles (Pd NPs) and CALB for tandem catalytic applications. Among the tested systems, the Pd&CALB cascade confined in COF‐OMe exhibited the highest efficiency in the dynamic kinetic resolution of primary amines (Figure 7a2). This enhancement was attributed to the interfacial activation mechanism of CALB, in which the hydrophobic microenvironment provided by COF‐OMe promoted the opening of the lid domain of CALB, thus exposing its active site. In addition, the hydrophobicity of COF‐OMe facilitated the dispersion and stabilization of Pd NPs, further contributing to the improved catalytic performance. In a separate study, Hao et al. designed a photothermally responsive COF (EP‐NKCOF‐73) by incorporating the azo group to regulate the local catalytic temperature of the encapsulated lipase under light irradiation (Figure 7b1).^[^ ^108^ ^]^ Lipase was covalently immobilized on the pore surface of EP‐NKCOF‐73. Upon solar light exposure, the local temperature of the lipase@EP‐NKCOF‐73 system significantly increased, surpassing that observed in both the free lipase and blank control systems (Figure 7b2). This localized photothermal effect was conducive to accelerating reaction kinetics and enhancing substrate diffusion. As a result, lipase@EP‐NKCOF‐73 demonstrated superior catalytic activity compared to its free counterpart (Figure 7b3).

**Figure 8:**
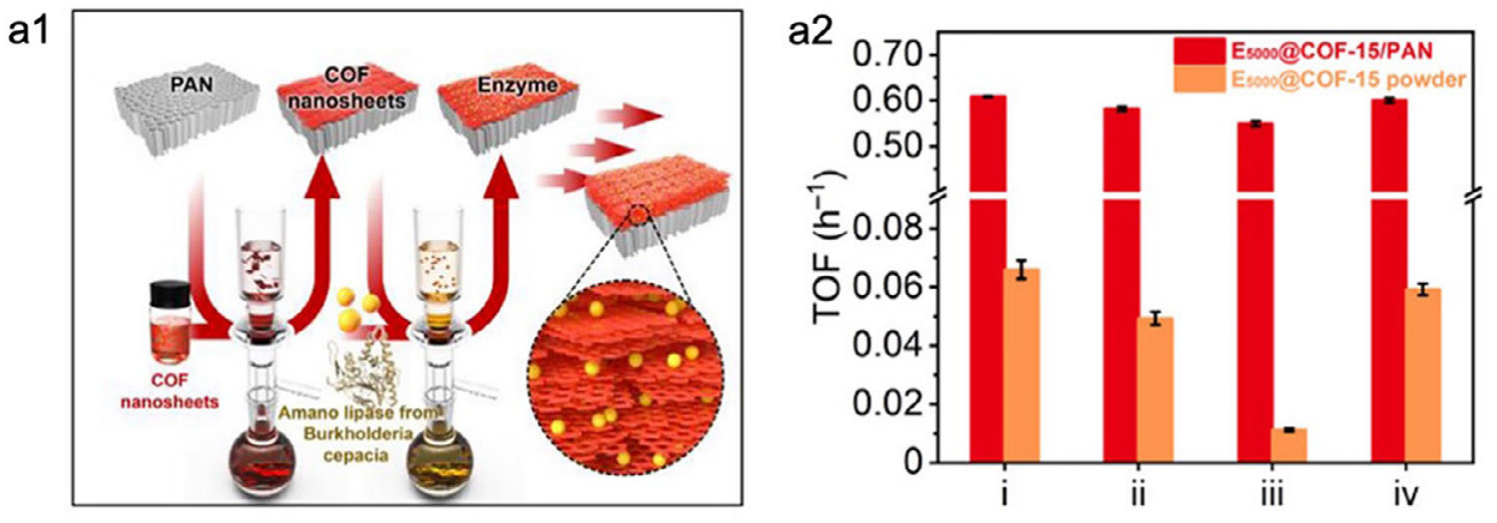
a1) Schematic illustration of enzyme immobilization within COF nanosheet‐based membranes for constructing efficient bioreactors. a2) Comparing the catalytic performance of enzymes@COF‐15/PAN and enzymes@COF‐15 powder as catalyzing different substrates. Reproduced with permission.^[^ ^109^ ^]^ Copyright 2025, Wiley‐VCH.

In addition to tuning the polarity and photothermal properties of COFs, confining enzymes within 2D COF membranes has emerged as a promising strategy for enhancing enzymatic performance. For instance, in the work by Chen et al., ultrathin COF nanosheets were assembled into a membrane bioreactor (COF‐15/PAN) for the co‐immobilization of GOx and HRP (**Figure** **8**a1).^[^ ^109^ ^]^ The well‐oriented lamellar structure enabled the co‐localization and alignment of enzymes, thus decreasing substrate diffusion pathways and facilitating efficient cascade catalysis. Therefore, the enzymes@COF‐15/PAN membrane exhibited significantly higher turnover frequencies (TOFs) for all tested substrates (i–iv) compared to the COF‐15 powder‐based system (Figure 8a2). Moreover, the nanoconfined planar channels of the COF membrane provided a mildly hydrophilic and size‐selective microenvironment, which is favourable to preserve enzyme stability and minimize enzyme leaching. Collectively, these advantages endowed the membrane‐based biocatalytic system with superior catalytic efficiency, operational stability, and reusability, outperforming conventional immobilized enzyme systems.

## 4 HOFs‐Based Biocomposites

### 4.1 Insight into Enzyme Encapsulation in HOFs

HOFs are novel porous crystalline materials constructed from organic tectons via hydrogen bonding interactions, further stabilized by van der Waals forces and π–π stacking, featuring high structural regularity, mild synthesis conditions, and dynamic reversibility.^[^ ^47^, ^48^, ^110^, ^111^, ^112^ ^]^ Compared to the non‐physiological synthesis environments of some MOFs and COFs, the gentle preparation of HOFs matrices can endow them with outstanding biocompatibility for in situ loading of biomacromolecule guests.^[^ ^60^, ^61^, ^62^ ^]^ Moreover, using HOFs as a platform can enhance the safe applications in biological fields due to their metal‐free composition. These benefits make HOFs an excellent platform to immobilize enzymes, which significantly facilitates enzymes to be stably, durably, and efficiently applied in the complicated and volatile practical operation environments.^[^ ^45^, ^46^ ^]^ The in‐situ encapsulation of enzymes in HOFs can be achieved through a co‐coprecipitation process, in which enzyme molecules are introduced into the self‐assembly system, allowing them to become embedded within the porous network as it forms.^[^ ^60^, ^61^, ^62^ ^]^ BioHOF‐1^[^ ^60^ ^]^ and HOF‐101,^[^ ^61^, ^62^ ^]^ featuring distinct hydrogen‐bonding motifs (N─H···O and O─H···O, respectively), represent pioneering HOFs employed for the encapsulation of single‐ and multi‐enzyme systems. The obtained immobilized enzymes in BioHOF‐1 and HOF‐101 exhibited superior stability across a wide pH range and improved catalytic performance, respectively, in comparison with those encapsulated in ZIF‐8. The causes may be that the poor stability of ZIF‐8 under acidic conditions^[^ ^113^ ^]^ and the substrate diffusion resistance within ZIF‐8 (pore aperture: 0.34 nm^[^ ^67^ ^]^), respectively.

**Figure 9:**
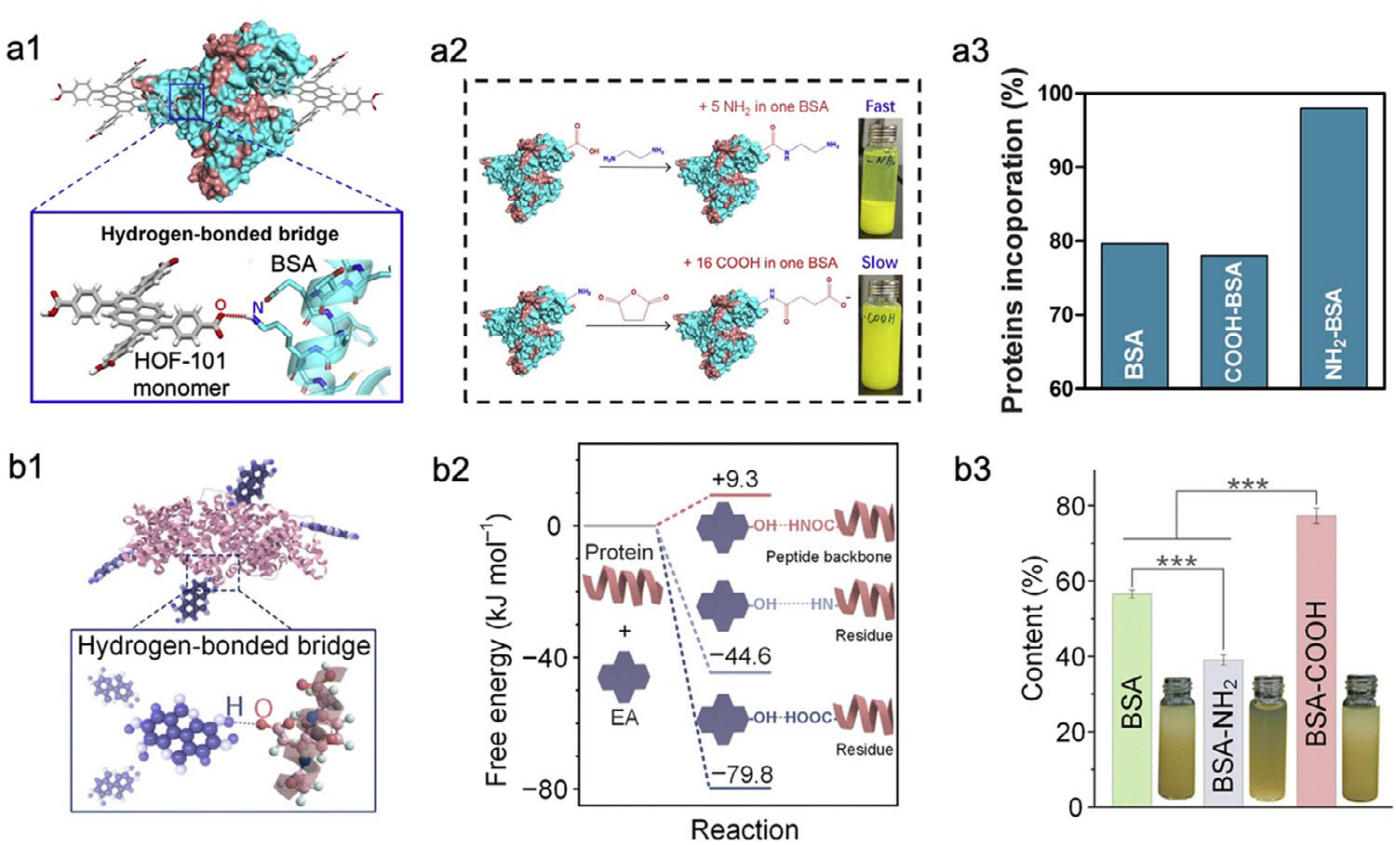
Representative examples of encapsulating enzymes within HOFs constructed by a single organic tecton. a1) Schematic illustration of the hydrogen‐bonded interaction between N–H residues of BSA and the organic linker of HOF‐101. a2) Schematic illustration of the assembly differences of COOH‐BSA, and NH_2_‐BSA within HOF‐101. a3) The encapsulation efficiency of BSA, COOH‐BSA, and NH_2_‐BSA within HOF‐101. Reproduced with permission.^[^ ^61^ ^]^ Copyright 2021, Elsevier. b1) Schematic illustration of the hydrogen‐bonded interaction between BSA and EA. b2) Comparison of free energy changes between EA and proteins with different hydrogen bonding models. b3) The encapsulation efficiency of BSA, COOH‐BSA, and NH_2_‐BSA within SPF. Reproduced with permission.^[^ ^114^ ^]^ Copyright 2024, The Royal Society of Chemistry.

**Figure 10:**
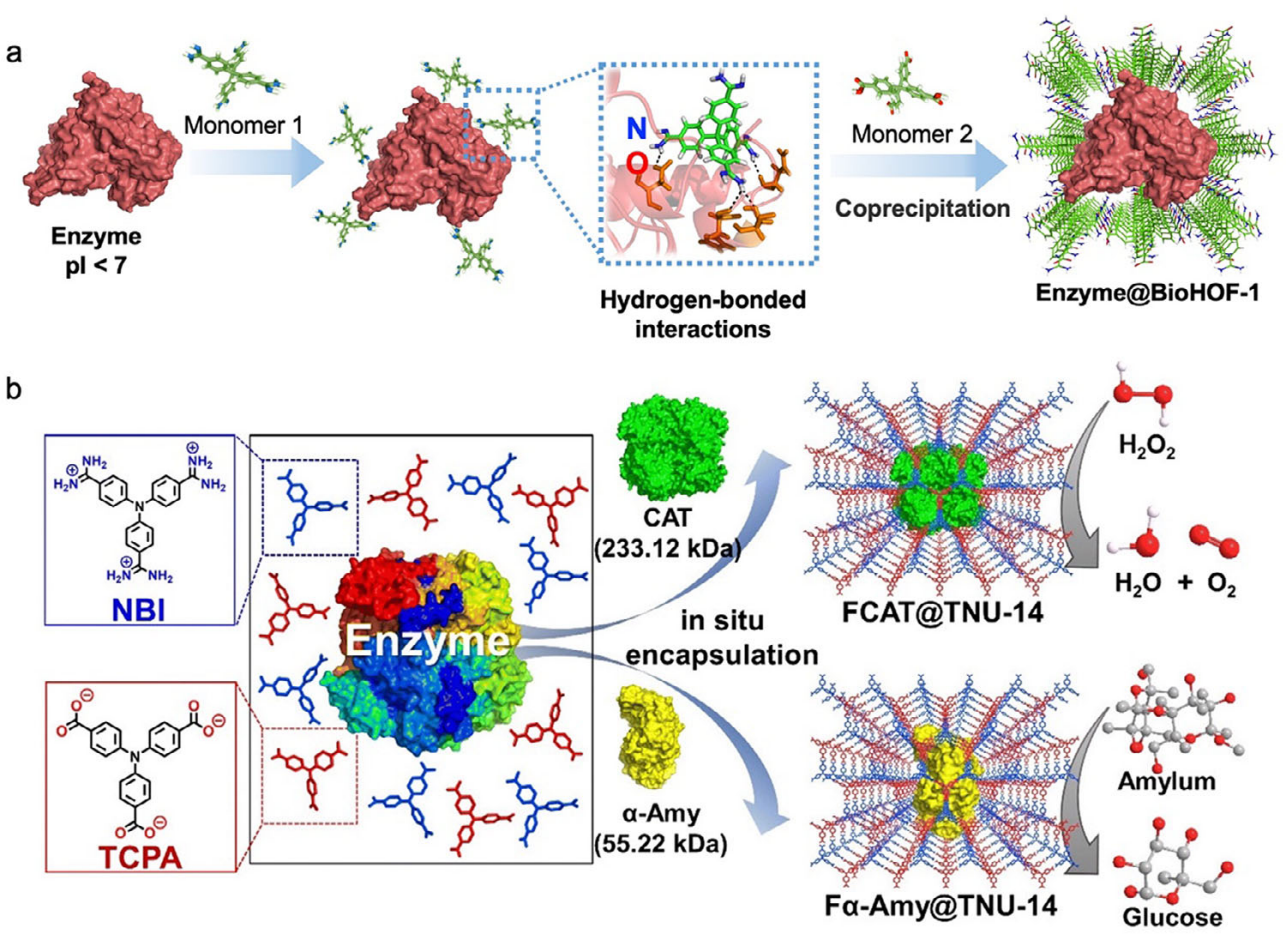
Representative examples of encapsulating enzymes within HOFs constructed by two organic tectons. a) Schematic illustration of the process of encapsulating acidic proteins within BioHOF‐101. pI, isoelectric point. Reproduced with permission.^[^ ^116^ ^]^ Copyright 2023, Wiley‐VCH. b) Schematic illustration of encapsulating different enzymes within TNU‐14. Reproduced with permission.^[^ ^115^ ^]^ Copyright 2025, Royal Society of Chemistry.

The encapsulation of enzymes within HOFs is highly dependent on their surface charge, as the *in‐situ* co‐crystallization process proceeds through charge‐mediated interactions that dictate the enzyme–framework association and spatial distribution. HOF‐101 represents a typical example of HOFs assembled from a single organic tecton mainly through intermolecular hydrogen‐bonding interactions among carboxyl groups. The encapsulation of basic proteins within HOF‐101 is more efficient than that of acidic counterparts, owing to favorable electrostatically driven hydrogen‐bonding interactions between the nitrigen‐rich surfaces of basic proteins and the carboxylate units of the framework (**Figure** **9**a).^[^ ^61^ ^]^ A comparable behavior was found in the ellagic acid (EA)‐assembled HOF (SPF), where the framework constructed via hydroxyl‐mediated hydrogen‐bonding interactions, preferentially encapsulates acidic proteins. This preference arises from the stronger O─H···O interactions between the carboxylate residues of acidic proteins and EA, whereas hydrogen bonds involving the amine residues of basic proteins are intrinsically weaker and less stable (Figure 9b).^[^ ^114^ ^]^ In contrast, dual‐ligand HOFs (e.g., BioHOF‐1^[^ ^60^ ^]^ and TNU‐14^[^ ^115^ ^]^) display minimal dependence on enzyme surface charge, allowing for the encapsulation of proteins with diverse electrostatic properties (**Figure** **10**). Specifically, the protein (e.g., acidic protein) can first be pre‐mixed with one type of ligands (e.g., amino‐functionalized ligand) that can form favorable hydrogen bonds with its surface residues, followed by the addition of the second ligand to trigger co‐precipitation and HOF assembly (Figure 10a).

Although HOFs offer clear benefits for enzyme immobilization, the recovered enzymatic activity after immobilization is still lower compared to the free native enzymes.^[^ ^60^, ^61^, ^62^ ^]^ These results can be attributed to the formation of non‐covalent interactions between the residues on the enzyme surface and the building blocks of HOFs, which may obstruct the substrate access to enzymes and reduce their conformation flexibility.

### 4.2 Topology Tuning of HOFs

**Figure 11:**
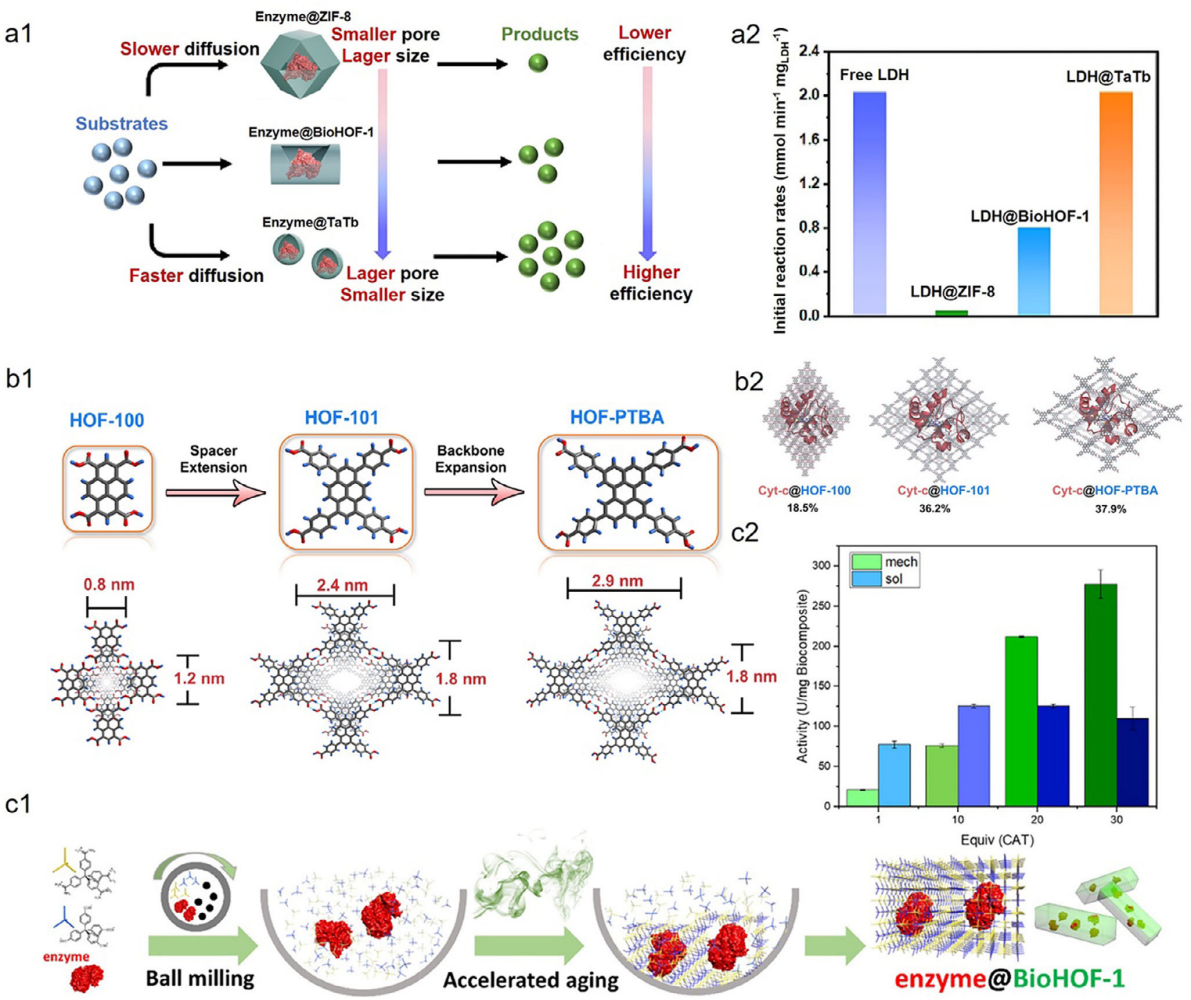
Representative examples of enhancing the performance of HOFs‐based biocomposites via tuning the topology of HOFs. a1) Schematic illustration of the biocatalysis advantages of TaTb HOF biocatalyst with increased pore size over ZIF‐8 and BioHOF‐1. Ta, 4,4′,4″‐tris(benzimidazol‐1‐ylmethyl)triphenylamine; Tb, 1,3,5‐tris(4‐carboxyphenyl)benzene. a2) Initial reaction rates of free LDH, LDH@ZIF‐8, LDH@BioHOF‐1, and LDH@TaTb. Reproduced with permission.^[^ ^117^ ^]^ Copyright 2023, Elsevier. b1) Schematic illustration of expanding the organic tecton backbone of HOFs. b2) Immobilization efficiency of Cyt c in HOF‐PTBA, HOF‐100, and HOF‐101. Reproduced with permission.^[^ ^120^ ^]^ Copyright 2024, Royal Society of Chemistry. c1) Schematic illustration of vibrational dry milling synthesis of enzyme@BioHOF‐1. c2) Specific activity of enzyme@BioHOF‐1 and the corresponding synthesized in solution. Reproduced with permission.^[^ ^124^ ^]^ Copyright 2025, Wiley‐VCH.

Enlarging the pore size of HOFs is conducive to promoting the diffusion of substrate, thus enhancing the catalytic performance of encapsulated enzymes. For instance, Li et al. designed a nano‐sized mesoporous HOF (TaTb nmHOF) for the in‐situ encapsulation of NADH‐dependent lactate dehydrogenase (LDH) to catalyze the bioconversion of pyruvate (**Figure** **11**a1).^[^ ^117^ ^]^ Compared with ZIF‐8 (0.34 nm) and BioHOF‐1 (1.2 nm), TaTb nmHOF exhibited a larger pore size of 2.4 nm, which facilitates the diffusion of bulky molecules such as NADH and pyruvate (Figure 11a1), while also being beneficial to prevent blockage of the active site of LDH. As a result, the catalytic efficiency of LDH@TaTb nmHOF was significantly higher than that of LDH@BioHOF‐1 and LDH@ZIF‐8 (Figure 11a2). Though the activity recovery of LDH@TaTb nmHOF was comparable to that of free LDH (Figure 11a2), the encapsulation of other enzymes (e.g., α‐amylase and HRP) in TaTb nmHOF resulted in lower activity recovery. Another strategy to enlarge the pore size of HOFs involves extending the length of the organic tectons. However, this can induce undesired network interpenetration or layer staggering, ultimately decreasing the effective pore size and compromising structural stability.^[^ ^118^, ^119^ ^]^ Zhao, Li, and co‐workers reported an ultrastable mesoporous HOF (HOF‐PTBA) based on a bulky π‐conjugated perylene backbone (Figure 11b1).^[^ ^120^ ^]^ Owing to its expanded 1D square channels (18 × 29 Å), HOF‐PTBA achieved a higher Cyt *c* encapsulation efficiency compared with HOF‐100 and HOF‐101 (Figure 11b2). Although enlarging the pore size of HOFs promotes mass transfer, improves enzyme encapsulation, and partially enhances the structural flexibility of confined enzymes, their catalytic activity recovery is sometimes still inferior to that of the free enzymes.^[^ ^117^, ^120^ ^]^ These results indicate that tuning the pore size of HOFs is not a universally effective strategy for enhancing the activity recovery of all encapsulated enzymes.

In solution, the rapid precipitation of HOF crystals often confines enzymes at the crystal surface or pore openings.^[^ ^60^, ^121^, ^122^, ^123^ ^]^ Such spatial crowding, combined with solvent molecules competing for hydrogen‐bonding sites on both the enzyme and the ligands, can lead to partial structural denaturation and restricted conformational dynamics of the enzyme.^[^ ^124^ ^]^ Therefore, Hafner et al. developed a vapor‐assisted mechanochemical synthesis for HOFs‐based biocomposites (Figure 11c1), in which the slow crystallization process favored the homogenous distribution of the enzyme throughout the HOF crystal.^[^ ^124^ ^]^ The resulting encapsulated enzyme displayed an improved protein loading and higher specific activity than their solution‐prepared counterparts (Figure 11c2). It is worth noting that, due to the slow crystallization kinetics and narrow processing window, this method tends to cause partial loss of enzymatic activity and poses challenges for large‐scale implementation. Despite these drawbacks, solvent‐free synthesis offers significant potential for advancing reticular chemistry‐based biocomposites, benefiting from its environmentally benign nature and facile operation.

### 4.3 Enzyme Surface Engineering

**Figure 12:**
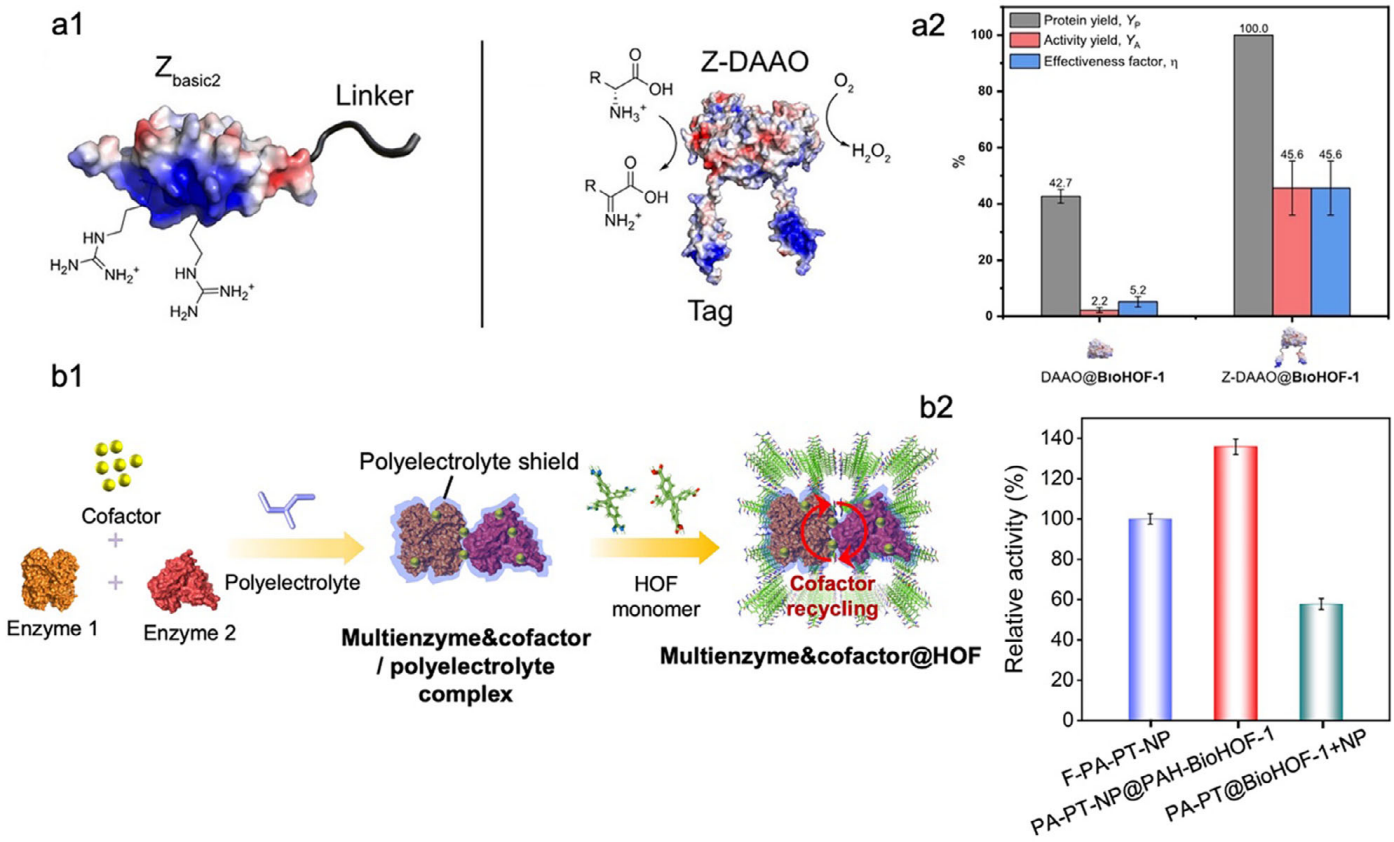
Representative examples of enhancing the performance of HOFs‐based biocomposites via engineering the enzyme surface. a1) Crystal structure of Z_basic2_ binding module (left) and Z‐DAAO (right). a2) Immobilization performance of DAAO@BioHOF‐1 and Z‐DAAO@BioHOF‐1. Reproduced with permission.^[^ ^121^ ^]^ Copyright 2022, Wiley‐VCH. b1) Schematic of the co‐encapsulation of multienzyme and NAD(P) cofactors into BioHOF‐1 via the assistance of PAH for several biocatalysis. b2) Relative cascade activity of the NAD‐dependent ADH&AmDH system at different forms, including free mixture, co‐encapsulation in PAH‐BioHOF‐1, and encapsulation in BioHOF‐1 (excluding NADH). Reproduced with permission.^[^ ^116^ ^]^ Copyright 2023, Wiley‐VCH.

Rationally designing a module capable of mediating enzyme–HOF interactions provides an effective strategy to improve enzyme activity recovery by reducing unfavorable interactions between the framework and the enzyme. Wied et al. engineered _D_‐amino acid oxidase (DAAO) by fusing a Z_basic2_ module to its N‐terminus, generating Z‐DAAO (**Figure** **12**a1).^[^ ^121^ ^]^ Owing to the NH_2_‐rich residues of the Z_basic2_ tag, which promote stronger hydrogen‐bonding interactions with the BioHOF‐1 matrix, Z‐DAAO@BioHOF‐1 exhibited a 20‐fold enhancement in catalytic activity compared with DAAO@BioHOF‐1 (Figure 12a2). Moreover, the immobilization efficiency of Z‐DAAO@BioHOF‐1 was 2.3‐fold higher than that of DAAO@BioHOF‐1, attributed to the additional binding sites provided by the Z_basic2_ module to BioHOF‐1 (Figure 12a2). These findings demonstrate that surface engineering of enzymes through the incorporation of residues capable of hydrogen bonding with HOF building blocks not only enhances activity recovery within the frameworks but also increases enzyme loading capacity. However, the catalytic activity of Z‐DAAO@BioHOF‐1 was still lower than that of free counterpart (Figure 12a2). The cause may be that, although the Z_basic2_ module could compete with DAAO for interactions with HOF, residual contacts between the framework and the enzyme persist, thus limiting full restoration of enzymatic activity. Additionally, the genetic engineering of enzymes displays complexity and lacks universality, and may compromise their intrinsic catalytic activity.

A more facile yet versatile strategy was reported by Liu et al., in which a poly(allylamine hydrochloride) (PAH) was employed to electrostatically coat onto the surfaces of enzymes against the adverse interactions of HOF (Figure 12b1).^[^ ^116^ ^]^ Meanwhile, NAD(P) cofactors were attached onto PAH through the electrostatic interactions (Figure 12b1). Afterwards, the co‐immobilized multienzyme and NAD(P) system was constructed within PAH‐BioHOF‐1 through hydrogen‐bonding and electrostatic interactions between PAH and BioHOF‐1 monomers (Figure 12b1). PAH in this work played two key roles, one in protecting enzymes to realize the maximum biocompatibility of HOFs and the other in tethering NAD(P) cofactors to make them along with enzymes to be encapsulated in HOFs. Using the NAD‐dependent alcohol dehydrogenase (ADH) and amine dehydrogenase (AmDH) cascade as an example, therefore, the co‐encapsulated catalytic systems in PAH‐BioHOF‐1 exhibited significantly enhanced overall catalytic activity compared to the corresponding system in BioHOF‐1, and even over outperformed the free counterpart (Figure 12b2). The increased cascade performance was attributed to the proximity effect of two enzymes and enhanced cofactor affinity for the enzymes, apart from the excellent enzyme activity recovery. Notably, the local cofactor pool formed through the electrostatic attraction of polyelectrolyte can be easily integrated into various reticular frameworks, demonstrating excellent versatile. Meanwhile, the pre‐protection strategy, which prevents the unfavorable interactions between reticular tectons and enzymes to preserve the native conformation of enzymes, exhibits substantial potential in designing biomimetic microenvironment in the construction of enzyme‐integrated MOFs/COFs/HOFs.

### 4.4 Enzyme‐HOFs Interface Design

**Figure 13:**
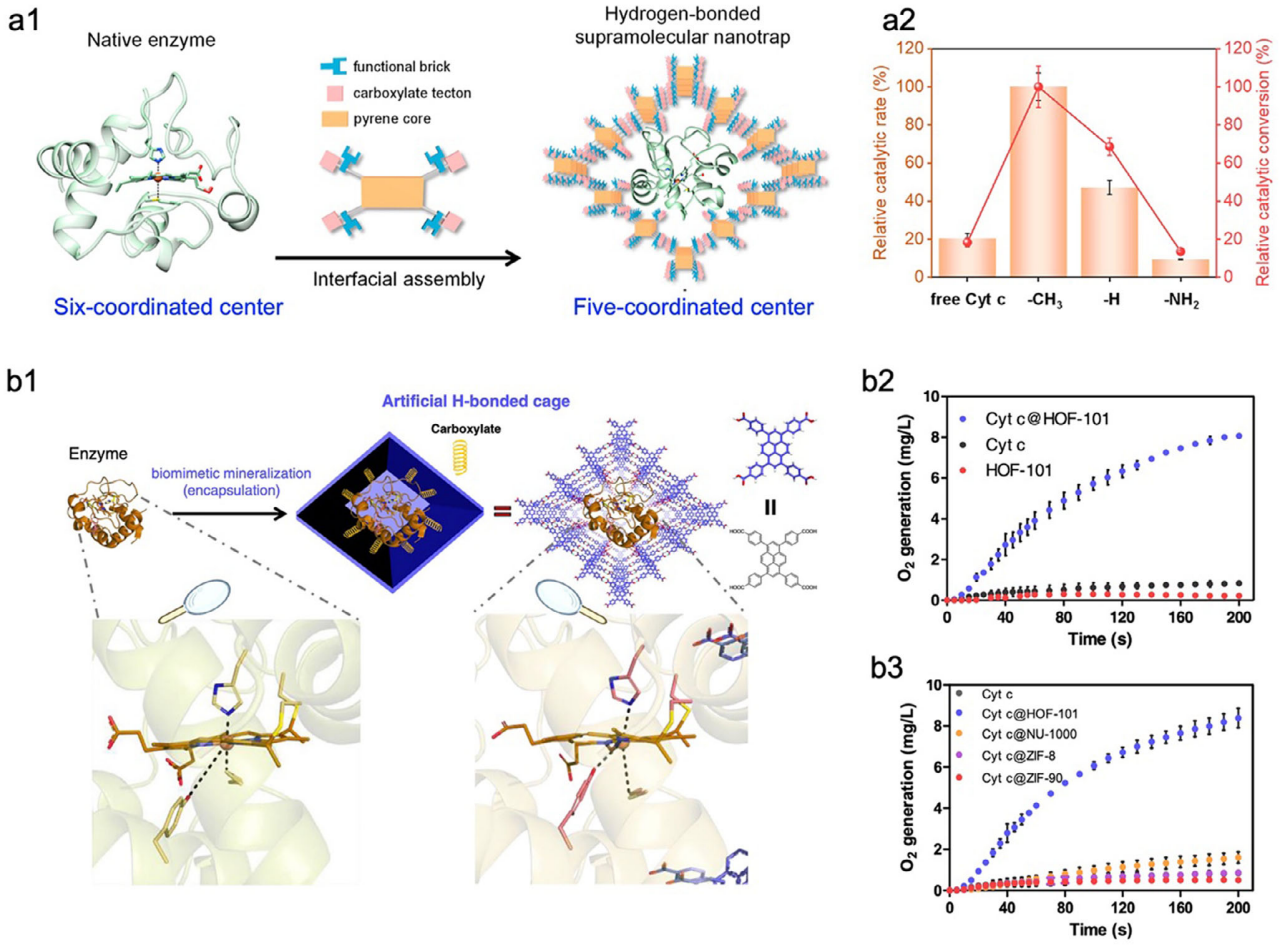
Representative examples of tailoring the performance of HOFs‐based biocomposites. a1) Schematic of encapsulating Cyt *c* within HOF‐101‐CH_3_ with a hydrophobic pore microenvironment by atomically precise position of functional struts onto the HOF tectons. a2) Comparison of the relative reaction rates and conversions of free Cyt *c*, Cyt *c*@HOF‐101‐CH_3_, Cyt *c*@HOF‐101‐H, and Cyt *c*@HOF‐101‐NH_2_, respectively. Reproduced with permission.^[^ ^125^ ^]^ Copyright 2024, American chemical Society. b1) Schematic of a functional H‐bonded nanocage for regulating the conformation of Cyt *c*. b2) Evaluating of the catalase‐like activity of Cyt *c*@HOF‐101, Cyt *c*, and HOF‐101. b3) Comparing the catalase‐like activity among Cyt *c*@HOF‐101, Cyt *c*, Cyt *c*@NU‐1000, Cyt *c*@ZIF‐8, and Cyt *c*@ ZIF‐90. Reproduced with permission.^[^ ^126^ ^]^ Copyright 2022 Springer nature.

Although maintaining the native conformation of enzymes is beneficial for improving their activity retention within framework materials, optimizing the conformation of enzymes via a precise engineering design may lead to superior catalytic performance in certain cases. This approach primarily relies on the regulation of the bio‐nano interface. Given the strong interfacial interaction between the HOF pore wall of the nanotrap and the surface residues of the enzyme, Huang et al. functionalized the HOF‐101 tecton with two different groups, including the methyl (─CH_3_) and amino struts (─NH_2_), to create HOF‐101s with hydrophobic and hydrophilic microenvironments for in situ encapsulating Cyt *c*, respectively.^[^ ^125^ ^]^ Compared to the native Cyt *c*, the coordinated state of active centre iron (Fe) in Cyt *c*@HOF‐101‐CH_3_ decreased to five from six (**Figure** **13**a1), resulting from the strong hydrophobic interaction between the hydrophobic surface of the embedded Cyt *c* and the ─CH_3_ struts led to the dissociation of the original axial coordination of Fe and M80, which benefits the catalytic bonding of the ingoing substrate in an axial direction. Therefore, a 4.9‐fold enhancement in catalytic activity was observed for Cyt *c*@HOF‐101‐CH_3_ compared to free Cyt *c* (Figure 13a2). Of specific note, the pristine HOF‐101 could also offer a relatively hydrophobic interface environment, thus resulting in a superior catalytic performance over the free Cyt *c* (Figure 13a2). In contrast, the hydrophilic nanotrap of HOF‐101‐NH_2_ is unfavorable for the exposure of the heme center in the hydrophobic pocket of Cyt *c* and even compromised the enzyme activity (Figure 13a2). Furthermore, the changed conformation of an enzyme via the H‐boned nano‐biointerface could also generate non‐native biocatalytic activity. Chen et al. found that Cyt *c* encapsulated within HOF‐101 exhibited catalase (CAT)‐like activity, which was attributed to the formation of a CAT‐like conformation characterized by a high spin, five‐coordinated heme center (Figure 13b1).^[^ ^126^ ^]^ As mentioned above, the native Cyt *c* with the low spin, hexa‐coordinated heme cannot directly transfer H_2_O_2_ to O_2_ (Figure 13b2). Moreover, Cyt *c* encapsulated in common MOFs (i.e., NU‐1000, ZIF‐8, and ZIF‐90) exhibited no detectable catalytic activity toward H_2_O_2_ (Figure 13b3). This work highlights the unique advantage of HOFs in modulating enzyme conformation, which is rarely observed/reported enzyme‐MOFs or ‐COFs systems.

## 5 Conclusion and Future Perspectives

Reticular frameworks offer remarkable opportunities to enhance biocatalysis by enabling precise control over enzymatic reaction behavior through structural modulation, interfacial design, and protein surface engineering. Moreover, the modularity of reticular chemistry enables the incorporation of tailored functionalities (e.g., photothermal responsiveness and nanozyme performance), which expands the utility of these systems beyond simple immobilization platforms toward multifunctional, biomimetic architectures. Despite these, the distinct structural and synthetic properties as well as interfacial microenvironments of MOFs, COFs, and HOFs lead to important differences in strategy implementation. MOFs offer high crystallinity and structural tunability with relatively mild synthesis conditions, making them well‐suited for post‐synthetic conformational control and dynamic defect engineering. Moreover, MOFs allow precise structural modulation through metal node coordination and linker functionalization, enabling control of active‐site proximity and electron transfer but sometimes imposing rigid confinement that may distort flexible enzymes. COFs often require harsher synthesis conditions, necessitating pre‐protection strategies or the development of biocompatible polymerization methods for enzyme encapsulation. Additionally, COFs, built from covalent linkages, offer a chemically stable and designable interface, suitable for post‐synthetic modification and introduction of hydrophilic/hydrophobic domains, thus finely tuning enzyme–substrate interactions. HOFs, in contrast, rely on hydrogen‐bonded and relatively dynamic networks, which enable gentle encapsulation and adaptive interfacial contact. This flexibility minimizes structural strain on the enzyme and allows reversible modulation of catalytic activity under mild conditions. However, HOFs suffer from relatively weaker mechanical stability and less precise framework control. These distinctions not only dictate how enzymes are incorporated but also dictate the mechanisms through which catalytic enhancement is achieved. Quantitatively, these differences are reflected in variations of enzyme loading capacity, retained activity, and apparent kinetic parameters (Table 1).

Despite these advances in nanobiocomplexes based on reticular chemistry, several critical challenges remain:

1) The unpredictable nature of nano–bio interfacial interactions limits rational design, especially in multienzyme systems where conformational crosstalk and dynamic regulation are essential for cascade catalysis.
2) Although standard approaches for encapsulating enzymes within COFs^[^ ^96^ ^]^ and HOFs^[^ ^127^ ^]^ have been reported, the reticular frameworks employed in these protocols have largely been restricted to a few specific structural types (e.g., COF‐42/43, NKCOF‐98/99, and HOF‐101), leading to challenges in tailoring biocatalysis performance using other COFs and HOFs as the platform. The causes may arise from the intrinsic synthetic sensitivities of COFs and the diverse hydrogen‐bonding motifs and stacking arrangements of HOFs.
3) Encapsulation of bulky enzymes or multi‐enzyme complexes remains nontrivial, constrained by limited pore accessibility, non‐uniform spatial organization, and diffusion bottlenecks.
4) Lack of predictive models for designing optimal interfaces tailored to specific enzyme–substrate systems.

To address these limitations, we envision several promising directions:

1) Integrate high‐resolution *in‐situ*/operando characterization technologies (e.g., cryo‐electron microscopy,^[^ ^128^ ^]^ solid‐state nuclear magnetic resonance spectroscopy,^[^ ^70^ ^]^ neutron diffraction^[^ ^129^ ^]^) with molecular simulations to guide the design of enzyme–framework interfaces at the atomic level, enabling better control over conformation, orientation, and activity.
2) Employing modular or interfacial synthesis approaches may provide better control over nucleation and crystal growth, thus enhancing the reproducibility, scalability, and biocompatibility of various COFs‐ and HOFs‐based enzyme encapsulation systems.
3) Spatial engineering of reticular framework architectures using anisotropic or modular building blocks to create multi‐compartmentalized, size‐tunable, and directionally aligned systems for co‐localizing complex enzyme cascades.
4) Integration with machine learning to navigate the vast design space of framework structures^[^ ^130^, ^131^ ^]^ and enzyme candidates will accelerate the discovery of next‐generation hybrid biocatalysts.

In conclusion, reticular frameworks are transitioning from passive supports to active participants in biocatalysis, supporting unprecedented levels of control over enzyme function. Continued interdisciplinary innovation at the interface of materials science, protein engineering, and systems chemistry will be crucial for unlocking their full potential in catalysis, synthetic biology, and therapeutic applications.

## Conflict of Interest

The authors declare no conflict of interest.
